# Parkinson's disease uncovers an underlying sensitivity of subthalamic nucleus neurons to beta-frequency cortical input *in vivo*

**DOI:** 10.1016/j.nbd.2020.105119

**Published:** 2020-12

**Authors:** Magdalena K. Baaske, Eszter Kormann, Abbey B. Holt, Alessandro Gulberti, Colin G. McNamara, Monika Pötter-Nerger, Manfred Westphal, Andreas K. Engel, Wolfgang Hamel, Peter Brown, Christian K.E. Moll, Andrew Sharott

**Affiliations:** a Medical Research Council Brain Network Dynamics Unit, Nuffield Department of Clinical Neurosciences, University of Oxford, Mansfield Road, Oxford OX1 3TH, UK; b Department of Neurology, University of Lübeck, 23538 Lübeck, Germany; c Institute of Neurogenetics, University of Lübeck, 23538 Lübeck, Germany; d Department of Neurophysiology and Pathophysiology, University Medical Center Hamburg-Eppendorf, 20246 Hamburg, Germany; e Department of Neurology, University Medical Center Hamburg-Eppendorf, 20246 Hamburg, Germany; f Department of Neurosurgery, University Medical Center Hamburg-Eppendorf, 20246 Hamburg, Germany

**Keywords:** Cortex, Beta oscillation, Parkinson's disease, STN neuron, Synchronisation

## Abstract

Abnormally sustained beta-frequency synchronisation between the motor cortex and subthalamic nucleus (STN) is associated with motor symptoms in Parkinson's disease (PD). It is currently unclear whether STN neurons have a preference for beta-frequency input (12-35 Hz), rather than cortical input at other frequencies, and how such a preference would arise following dopamine depletion. To address this question, we combined analysis of cortical and STN recordings from awake human PD patients undergoing deep brain stimulation surgery with recordings of identified STN neurons in anaesthetised rats. In these patients, we demonstrate that a subset of putative STN neurons is strongly and selectively sensitive to magnitude fluctuations of cortical beta oscillations over time, linearly increasing their phase-locking strength with respect to the full range of instantaneous amplitude in the beta-frequency range. In rats, we probed the frequency response of STN neurons in the cortico-basal-ganglia-network more precisely, by recording spikes evoked by short bursts of cortical stimulation with variable frequency (4-40 Hz) and constant amplitude. In both healthy and dopamine-depleted rats, only beta-frequency stimulation led to a progressive reduction in the variability of spike timing through the stimulation train. This suggests, that the interval of beta-frequency input provides an optimal window for eliciting the next spike with high fidelity. We hypothesize, that abnormal activation of the indirect pathway, via dopamine depletion and/or cortical stimulation, could trigger an underlying sensitivity of the STN microcircuit to beta-frequency input.

## Highlights

• STN-neurons are selectively entrained to cortical beta oscillations in PD patients.
• Phase-locking of STN-neurons is linearly dependent on oscillation magnitude.
• Beta bursts in LFP/EEG are accompanied by transient synchronisation of STN spiking.
• STN neurons are selectively entrained to cortical beta stimulation in rats.
• Beta-selectivity of STN neurons is present in control and dopamine-depleted rats.

## 1 Introduction

Abnormally synchronised oscillations at beta frequencies (12-35 Hz) characterise the cortico-basal ganglia network of patients with Parkinson's disease (PD) (Brittain et al., 2014) and animal models of PD (Bergman et al., 1994; Sharott et al., 2005). Moreover, they are reduced by both dopaminergic medication or deep brain stimulation (DBS) (Marsden et al., 2001; Levy et al., 2002; Williams et al., 2002; Kuhn et al., 2008). Whether or not beta oscillations are causal in generating akinetic motor symptoms, they provide a useful biomarker of the disease (Zaidel et al., 2009; Stein and Bar-Gad, 2012; Brittain and Brown, 2014). This is evident in multiple studies that demonstrated positive correlations between metrics of beta oscillations and symptom severity (Kuhn et al., 2005; Kuhn et al., 2008; Kühn et al., 2009; Little et al., 2012; Sharott et al., 2014; Neumann et al., 2016; Sharott et al., 2018) and the utilisation of their temporal dynamics to drive effective closed-loop DBS (Little et al., 2013).

While these activities clearly extend across the entire network (Mallet et al., 2008a; Pasquereau and Turner, 2011; Brazhnik et al., 2016; Deffains et al., 2016; Sharott et al., 2017), the STN likely has a pivotal role (Deffains et al., 2016). This stems, partly, from its position as the only group of excitatory neurons within the basal ganglia network. STN neurons phase-lock to beta-frequency population oscillations (measured by EEG/ECoG and LFP recordings) in cortex as well as STN and other basal ganglia structures (Mallet et al., 2008a; Moran et al., 2008; Tachibana et al., 2011; Shimamoto et al., 2013; Sharott et al., 2018). Cortical oscillations lead those in the STN (Williams et al., 2002; Fogelson et al., 2006; Lalo et al., 2008; Sharott et al., 2018), suggesting that STN neurons are entrained by cortical beta oscillations. It remains to be elucidated whether this entrainment is due to intrinsic resonance, network dynamics or a combination of the two.

Like all basal ganglia neurons outside the striatum, STN neurons fire autonomously via a specific set of intrinsic mechanisms (Nakanishi et al., 1987; Bevan and Wilson, 1999). The pattern of spikes that results from this autonomous firing is then shaped by a variety of synaptic inputs. The most prominent inputs to STN neurons are excitatory synapses from pyramidal tract neurons in the frontal cortex, often referred to as the “hyperdirect” pathway (Nambu et al., 2002), and inhibitory synapses from the globus pallidus external segment (GPe) (Smith et al., 1998; Bevan et al., 2002). Input from the GPe can also be heavily influenced by cortical output through the cortically-driven striatal indirect pathway neurons (Kita and Kita, 2011; Zold et al., 2012). When isolated from their inputs, the autonomous firing of STN neurons consists of highly regular single spiking (Bevan and Wilson, 1999). Irregular firing, bursting and oscillation are thus the result of the synaptic inputs perturbing this resting firing pattern (Wilson, 2015). Such external perturbations in firing pattern are dependent on the timing of excitatory and inhibitory inputs relative to the intrinsic currents driving autonomous firing (Wilson, 2013, Wilson, 2015). Despite the convergence of both excitatory and inhibitory afferents on single neurons and rich oscillatory input from cortex (Murthy and Fetz, 1992, Murthy and Fetz, 1996), STN neurons do not display sustained oscillations or synchrony in the healthy brain (Bergman et al., 1994; Wilson, 2013; Deffains et al., 2016). A key question, therefore, is, whether the mechanism to synchronise preferentially with beta-frequency cortical input is always present, but is prevented by an active decorrelation mechanism that is lost in PD (Wilson, 2013).

Here, we demonstrate in human PD patients that STN neurons display beta-frequency selective phase-locking and temporal dynamics relative to the instantaneous amplitude of population oscillations. In anaesthetised rats, we then manipulated the frequency of cortical input to identified STN neurons. These experiments suggested that beta-frequency input is optimal for reducing the variance in STN spike timing in both control and parkinsonian animals. Together, our findings suggest that STN neurons are selectively sensitive to beta-frequency input.

## 2 Methods

### 2.1 Human data

#### 2.1.1 Patient information

To address the question of how the firing of STN neurons is related to ongoing population oscillations in humans we analysed neuronal activity from patients undergoing deep brain stimulation surgery targeting the STN. The study was conducted in the agreement with the Code of Ethics of the World Medical Association (Declaration of Helsinki, 1967) and was approved by the local ethical committee. All patients gave their written informed consent to participate in the study. All patients included in the study suffered from advanced Parkinson's disease and did not show cognitive impairment as reflected by the Mattis Dementia Rating Scale (clinical details are given in Supplementary Table 1). Dopamine agonists were replaced by levodopa >7 days and levodopa was paused on the day of surgery. The operation was conducted under local anaesthesia and systemic analgosedation with remifentanile (0.01–0.1 μg/kg/min) and paused during the procedure of microelectrode recordings. The detailed surgical procedure is described elsewhere (Hamel et al., 2003; Moll et al., 2014; Sharott et al., 2014). The stereotactic coordinates for the STN were 11–13 mm lateral to the midline, 1–3 mm inferior and 1–3 mm posterior to the mid-commissural point on both sides. The rostral and caudal borders of the STN were determined by electrophysiological criteria (i.e. increase in background noise, irregular tonic discharge pattern, oscillatory and burst patterns) (Sterio et al., 2002; Sharott et al., 2014).

#### 2.1.2 Data acquisition and recording setup

STN LFP and unit recordings were collected during the standard neuro-navigation procedure applied for clinical electrode placement. Microelectrode signals were performed in the BenGun configuration (MicroGuide and NeuroOmega, Alpha Omega, Nazareth, Israel) with 1–3 parallel electrodes. Each electrode was 2 mm from the central tip, which was aimed at the final target position. Microelectrode signals were amplified (x20000), band-pass filtered (300–6000 Hz) and digitized (sampling rate: 20 kHz or 44 kHz). Local field potentials obtained from the macro-tip 3 mm above the micro-tip (contact size ≈ 1 mm, impedance <1kΩ) were band-pass filtered between 1 and 300 Hz, amplified (x5000-10,000) and sampled at 1375 or 3005 Hz. All signals were referenced to the unisolated distal part of the guide tube. Simultaneous cortical recordings were made using either the electrocorticogram (ECoG) from the dura (approximately above dorsolateral prefrontal cortex) or the EEG positioned at Fz according to the international 10–20 system, both re-referenced to Pz. Referencing resulted in considerably cleaner signals, however, we cannot rule out that the EEG and ECoG signals are also influenced by the more caudal electrode. Both signals are thus likely to reflect activity from relatively widespread cortical areas. Fz was used in addition to ECoG, because ECoG was only available in 10/16 patients. LFPs were not recorded in two patients, which were therefore only included for analysis of cortical signals and units. Please note that macro tips were located in the STN or directly supra-STN in the thalamus (Nucleus ventralis oralis anterior of the thalamus (VOA)) or the zona incerta (ZI), thus LFPs probably originating from postsynaptic potentials from all these structures together. Limiting the analysis to LFPs located in the STN revealed no major differences so that for increasing n-numbers all available LFPs were included.

#### 2.1.3 Data selection and processing

Recordings had a variable length (109 +/− 77 s), and depending on recording quality, total recordings time and trajectories, the number of extracted single and multi-units varied between patients (18.88 +/− 9.67 units per patient). Spike detection and spike sorting was performed offline using Offline Sorter (Plexon Inc., Dallas, TX, USA). The threshold was set for each individual recording based on the distribution relative to the background noise, typically >4 SD. Single units were then extracted by a manual sorting procedure in 2D and 3D feature space based on several waveform parameters as principal components (as well as on signal energy, peak time and the presence of a trough in the auto-correlogram) (Sharott et al., 2014; Sharott et al., 2018). Units corresponding to the putative spiking activity of up to 3 neurons and thus not fulfilling the criteria for well isolated single unit activity were classified as MUA, but were clearly distinct from background activity (Sharott et al., 2018). The mean firing rate of included units was 37.62 Hz +/− 18.84 Hz (*n* = 302). Note that units classified as MUA (*n* = 173) had a slightly higher firing rate (39.72 +/− 20.24 Hz) than SUA (*n* = 129, 34.84 +/− 16.43 Hz) (Mann-Whitney *U* Test (MWUT), *p* = 0.04). Only stationary units that maintained a stable firing rate, calculated in 3 s bins, of >5 Hz for at least 30s were included in further analyses. Note that while both SUA and MUA were used to increase the total number of units analysed, similar results could be observed when only SUA was used.

All signals were down-sampled to a common sampling rate of 1000 Hz prior to all analyses.

#### 2.1.4 Spectral analysis

Prior to spectral analysis all signals were low-pass filtered at 200 Hz and notch-filtered at multiples of 50 Hz using digital zero-phase forward and backward filtering. Power spectra were calculated using a multi-taper approach with 3.5 slepian tapers and a frequency resolution of 0.5 Hz. Power spectra were normalised for 1/f by using a fitted pink noise spectrum for each recording (Feingold et al., 2015).

Oscillations in spike trains were detected by calculating the compensated power spectral density using Welch's method. The power spectral density was calculated using non-overlapping hanning windows with a frequency resolution of 0.49 Hz (NFFT 2048) and then normalised by a spectrum from 100 shuffled spike trains (Rivlin-Etzion et al., 2006). Confidence limits were calculated by using the compensated power in the range from 300 to 500 Hz. Significant peaks were detected at a significance level *p* < 0.05 which was corrected for the number of frequency bins in the range of interest between 0 and 100 Hz (Bonferroni corrected alpha significance level 0.05/206 bins: *p* < 2.43e-04). Example power spectra of STN unit spike trains and a distribution of beta power of spike trains showing significant (*n* = 64/302) and not significant oscillatory activity (*n* = 238/302) in their spike trains is shown in Supplementary Fig. 1. For reasons of simplification, STN units that show significant oscillatory activity based on the above described criteria will be referred to as ‘oscillatory units’ and units without significant oscillatory activity in their spike trains as ‘non-oscillatory units’. In this formalism we would like to highlight, that oscillatory units have significant higher oscillatory activity than non-oscillatory units, but that the latter may have weak oscillatory activity that is not captured by this threshold. For comparison with the phase-locking analysis we calculated the mean compensated power of the preferred frequency of phase-locking +/− 5 Hz and normalised it by the power between 300 and 500 Hz. Please note that only the oscillatory behaviour of significantly phase-locked units is analysed further, therefore n-numbers used for subsequent analyses are referring to significantly phase-locked STN-units.

#### 2.1.5 Phase-locking analysis

To determine the relationship between unit activity and ongoing oscillations detected in field potential recordings (EEG, ECoG, LFP) we performed a phase-locking analysis. The field signal was filtered in exponentially increasing frequency bands from 0 to 100 Hz using a 2nd order Butterworth filter using zero-phase forward and backward filtering. The instantaneous phase of the oscillation was extracted for each spike using the Hilbert transform, and the distribution of spike-field phases compared to a uniform distribution using the Rayleigh test (Berens, 2009). Significant locking of a unit to a population signal was defined using the Rayleigh test (*p* < 0.05). We then calculated the mean vector length as a measurement of phase-locking strength (Lachaux et al., 1999) using circular statistics (Berens, 2009). In this formalism the vector length varies inversely with the variance of instantaneous spike-field phases and can reach a value between 0 and 1. To control for the effect of differing number of spikes, (Vinck et al., 2010; Sharott et al., 2012) a z-score of vector length was calculated using 1000 ISI shuffled spike trains. A z-score over 2 was defined to represent significant phase-locking.

For all phase-locking analyses, n-numbers refer to unit-field pairs, unless otherwise stated. In contrast to EEG (Fz) and ECoG recordings (where one position was recorded for each hemisphere), for many patients multiple LFP electrodes were recorded coincidently with a unit. Therefore, when unit-LFP pairs were analysed, the vector lengths of all LFP-unit pairs for a given unit were averaged so that each unit constituted a unique sample.

#### 2.1.6 Magnitude dependent phase-locking

To determine whether STN unit phase-locking was dependent on oscillation magnitude, the phase-locking analysis was repeated for spikes occurring at select magnitudes of the field oscillation. The absolute values of the Hilbert transform were used to extract the instantaneous magnitude of a given oscillation. The distribution of magnitude values at spike time occurrence was divided into deciles, where the 1st decile is defined by the magnitude from 0 to the border of decile 1 (i.e. 1st decile is from 0 to 10% of the spike-time magnitude distribution, each part contains 1/10 of the magnitude distribution values, schematic illustration in Fig. 2A). The magnitude distribution was created based on values only occurring at spike times, which led to equal numbers of spikes in each decile. In this formalism, low magnitude values of the ongoing oscillation correspond to the 1st decile and highest magnitude values to the 10th decile.

We first analysed every available unit-field pair in frequency bands with exponentially increasing widths between 0 and 100 Hz. To gain a deeper understanding of this analysis in the beta-frequency range we repeated the analysis for significantly phase-locked units in a single beta-frequency band. Because peak beta frequencies could differ between patients and units, we selected the centre frequency between 12 and 40 Hz with the lowest Rayleigh statistic *p*-value for each recording. The magnitude threshold at which a given unit became phase-locked was defined as the lowest decile at which the z-score exceeded and maintained a value of >2 until the 10th decile.

#### 2.1.7 Correlation analysis

We calculated Pearson's correlation coefficient between the vector length and the decile number for each filter frequency. Values with a Cook's distance >1 were excluded prior to the correlation analysis (Cook, 1977). For characterizing the type of the correlation in the beta-frequency range we repeated the correlation analysis with the mean magnitude in each decile, normalised by the mean magnitude of the highest decile. In the result section we will refer to this analysis as ‘magnitude-correlation’ and unit-field pairs showing a significant positive correlation between mean magnitude at deciles and phase-locking strength will be referred to as ‘magnitude-correlated’ units.

#### 2.1.8 Beta burst analysis

To analyse phase-locking during beta bursts, field signals (LFP, ECoG, EEG) were band-pass filtered using 11 overlapping frequency bins from 10 to 40 Hz (5 Hz wide, 2.5 Hz overlap, 2nd order Butterworth filter with digital zero phase forward and backward filtering). For each STN-unit – field pair (Fz *n* = 280, ECoG *n* = 186, LFP *n* = 248) we first repeated the phase-locking analysis in the above mentioned frequency bins. For determining the frequency of preferred locking for each significantly phase-locked STN-unit – field pair (here, Fz *n* = 148, ECoG *n* = 107, LFP *n* = 158), the filtered frequency with the lowest p-value of the Rayleigh statistic was chosen. We then applied the previously described threshold of the 75th percentile of the magnitude of the filtered signal and detected continuous episodes of elevated beta power above that threshold (Tinkhauser et al., 2017b; Tinkhauser et al., 2017a; Tinkhauser et al., 2018). Only beta bursts with a minimum duration of 3 cycle lengths were included in the analysis. We then repeated the phase-locking analysis of all spikes occurring within 1 cycle wide bins before, during, and after the beta burst. Vector length was only calculated when a minimum of 20 spikes occurred across all the cycles at a given position relative to the burst (e.g, all cycles around the peak of the beta burst).

We next addressed the hypothesis that magnitude-correlated and uncorrelated units are entrained differently during beta bursts. For this, we used the classification based on the correlation analysis described above. This allowed us to distinguish units that showed increasing phase-locking strength with increasing field magnitude, ‘magnitude-correlated’, as described previously) and performed statistics on a group of STN-unit-LFP/ECoG/LFP pairs, which were positive correlated with the magnitude (Fig. 6) and a group that was uncorrelated (Supplementary Fig. 5).

#### 2.1.9 Rate analysis

The mean firing rate in each magnitude decile and in each bin relative to the beta burst was calculated to determine whether increases in phase-locking strength were related to rate changes.

### 2.2 Rat data

#### 2.2.1 Juxtacellular recordings of STN neurons and cortical stimulation in control and 6-OHDA lesioned rats

Experiments were carried out on 5 healthy control male rats and 9 6-OHDA hemilesioned male rats (adult Sprague-Dawley rats, Charles River) (190-200 g) under general anaesthesia and were conducted in accordance with the Animals Act (Scientific Procedures, 1986, United Kingdom) and the Society for Neuroscience Policies on the Use of Animals in Neuroscience Research.

#### 2.2.2 Induction of unilateral 6-hydroxydopamine (6-OHDA) hemi-lesions

Unilateral hemi-lesions of dopaminergic neurons in the substantia nigra pars compacta were induced as described previously (Magill et al., 2001). Briefly, animals were anesthetised with isoflurane (4% *v*/v isoflurane O2), and 1 μl (6 mg/ml) of 6-OHDA-toxin solution was injected over 10 min with a glass pipette (diameter 18 μm) through a small burr hole either in the medial forebrain bundle (4.1 mm posterior of bregma, 1.4 mm lateral to the midline, 7.9 mm ventral to the dura) (2 rats) or in the substantia nigra pars compacta (4.5 mm posterior of bregma, 1.2 mm lateral to the midline and 7.9 mm ventral to the dura) (7 rats). 25 min prior to this desipramine (25 mg/kg, Sigma, Poole, UK) was injected i.p. to protect noradrenergic neurons from cell death. Electrophysiological recordings were conducted at least 2 weeks or later post lesioning. Successful lesions were confirmed on the same sections by a significant reduction in Thyrosine-hydroxylase (TH) immune-reactivity in the striatum of the lesioned hemisphere in comparison to the intact side.

#### 2.2.3 Electrophysiological recordings and juxtacellular labelling

Electrophysiological recordings in the STN were performed under general anaesthesia with induction by isoflurane (4% v/v isoflurane in O2) and were maintained with ketamine (30 mg/kg i.p., Willows Francis) and xylazine (3 mg/kg, i.p. Bayer). Body temperature, heart rate and peripheral reflexes were monitored throughout. Single unit activity and LFPs were recorded using a glass electrode (tip diameter 1.5–2.5 μm, impedance measured in situ 10–30 MΩ) filled with 0.5 M NaCl solution and Neurobiotin (1.5% *w*/*v*, Vector Laboratories) targeting the STN (Magill et al., 2004). Signals were analogue amplified (x10, Axoprobe-1A amplifier (Molecular Devices), further amplified (x100, NL-106 AC-DC Amp, Digitimer) and sampled at 16.6 kHz using a Power 1401 Analog-Digital converter and Spike 2 acquisition and analysis software (Version 7.2, Cambridge Electronic Design). Unit activity was band-pass filtered between 300 and 5000 Hz (DPA-2Fs filter/amplifier, Digitimer) and LFPs were low-pass filtered at 2000 Hz (NL125 filters, Digitimer). For verification of exact recording positions a subset of putative STN-neurons were juxtacellularly labelled (Pinault, 1996; Magill et al., 2000) with Neurobiotin. During this procedure positive current pulses (2–10 nA, 200 ms, 50% duty cycle) were applied until the neuron's spike train showed entrainment by the current. The location was then post hoc confirmed by localising the Neurobiotin filled cell bodies within the borders of STN using standard immunohistochemistry methods. The position of unlabelled neurons was reconstructed based on the position of labelled neurons recorded in the same animal (Nakamura et al., 2014).

#### 2.2.4 Electrical stimulation of the motor cortex

For cortical stimulation parallel, bipolar stimulating electrodes (constructed from nylon-coated stainless steel wires, California Fine Wire, tip diameter 100 μm, tip separation 150 μm, impedance 10 kΩ) were implanted in the primary motor cortex (M1, stereotactic coordinates: 4.2 mm anterior and 3.5 mm lateral to Bregma, (Watson and Paxinos, 1986)) ipsilateral to the nigrostriatal lesion (Magill et al., 2004). The depth of the cortical electrode (2.2 mm below the dura) corresponded approximately to the layer 5/6 of M1. Stimulation was performed in 12 blocks of 5 stimuli at 4, 10, 15, 20, 25, 30 and 40 Hz with a 0.3 ms square-wave pulse (constant-current isolator, A360D, World Precision Instruments). The inter-stimulus-train interval was ranging from 2 to 2.86 s depending on stimulation frequency. Stimulation amplitudes ranged from 75 to 1000 μA and were adjusted depending on strength of modulation of the evoked LFP response e.g. the lowest amplitude that caused a visible response in the STN LFP was chosen.

#### 2.2.5 Histology and imaging

Standard indirect immunofluorescence protocols were used to verify the recording position of Neurobiotin labelled somata in the STN and the success of dopaminergic terminal loss in the striatum (Magill et al., 2001; Mallet et al., 2012). Rats were given a lethal overdose of ketamine (150 mg/kg) and were transcardially perfused with 300 ml of 0.1% *w*/*v* glutaraldehyde and 4% w/v paraformaldehyde in 0.1 M phosphate buffer. Extracted brains were stored overnight in fixative solution at 4 °C. 50 μm coronal sections were used for staining and washed 3 × 10 min in Triton PBS (PBS containing 0.3% *v*/v Triton X-100, Sigma). For visualization of STN borders we performed an immune-staining for the marker forkhead box protein P2 (FoxP2) (Hontanilla et al., 1997; Campbell et al., 2009). Sections were first incubated overnight with Cy3-conjugated streptavidin (1:1000 dilution, Life technologies) and then incubated for 1 h in Triton PBS containing 10% v/v donkey serum (NDS, Jackson ImmunoResearch Laboratories) followed by incubation over night at 4 °C with the primary anti-body (Triton PBS containing 1% v/v NDS with goat anti-FoxP2 antibody 1:500, Santa Cruz). After washing in Triton PBS, sections were incubated with the secondary antibody Alexa 488 flurophor (raised in donkey, 1:1000, Life Technologies). Then the washed, mounted and cover-slipped (Vectashield, Vector Laboratories) sections were inspected with an epifluorescence microscope (Zeiss Acio Imager M.2) for final confirmation of recording positions.

For verification of successful dopaminergic lesions coronal sections anterior-posterior +/− 0.5 mm from bregma were stained with tyrosine hydroxylase as marker for dopaminergic terminals and neurons. For indirect immunofluorescence the same incubation protocol as described above was used. Tyrosine hydroxylase immunoreactions (primary antibody: Chicken anti-TH, 1:500 Abcam; secondary antibody: Alexa Fluor 488 1:1000, Life Technologies) were carried out in 3 or more striatal and at least 1 nigral section. Only brains with a clear difference in the brightness of the lesioned and intact striatum were classified as successful hemilesioned animals.

#### 2.2.6 Imaging

High resolution images of the confirmed STN neurons were taken using a confocal microscope (Zeiss LSM 880) equipped with 20 × 0.8NAPlan-Apochromat and 40 × 1.4NA Plan-Apochromat oil immersion objectives and Stereoinvestigator v11.0 software (MBF Biosciences). To capture the streptavidin and the FoxP2 immuno-reactivity the following lasers and filter-sets were used: Argon 488 nm laser for Alexafluor 488 (emission detection: 493–554 nm) and HeNe 543 nm for Cy3 (emission detection: 554–681 nm). Imaging was performed with 10× and 40× objectives. At 40×, a series of multiple-plane z-stacked images (optical section thickness 0.8 μm) were captured in order to show the exact location of the somatodendritic structure of the well-filled STN neurons. Labelled STN neurons were visualised with FoxP2 by showing images of 63× magnification using 1.46NA objective, optimum x,y sampling according to Nyquist, 135 × 135 × 17 μm field of view/stack height, maximum intensity projection of 18 z planes (total height 17 μm). Images were opened with Fiji software (Schindelin et al., 2012)). Both channels of the low magnification (20×) image were processed using ‘Enhance contrast’ (a linear contrast adjustment that automatically sets white and black points) set to 0.1% of pixels being saturated. The high magnification *Z*-stack images were flattened using standard deviation projection. Contrast were then enhanced with 0.8% saturation levels for the streptavidin channel.

The extent of DA lesion was evaluated using Zeiss Axio Imager M.2, equipped with Hamamatsu Flash 4.0 LT camera and Colibri 7 LED illumination. For assessing the extent of dopamine lesion, the whole coronal section was captured (±0.5 mm bregma) using Stereoinvestigator v11.0 software (MBF Biosciences) ‘Virtual Tissue’ scanning mode. Images were taken on a low magnification using a Plan-Apo 5× objective (numerical aperture 0.16). To image the TH signal, the AlexaFluor-488 channel was used (LED excitation at 475 nm, filter-set: 38). Once the whole-section scans were performed, images were exported to Image J software without any further image processing. A grid of 1000 × 1000 μm was randomly overlaid on the image of the whole section and the most dorsolateral square (top corner of both striata) was chosen for measuring the brightness of the lesioned and non-lesioned hemispheres. Matching control areas were chosen from the cortex above each striatum using the same method. Mean brightness levels were measured for all four squares. Each striatal brightness measure was then divided by the corresponding cortical brightness measure and were compared with each animal. Animals were considered lesioned if there was more than 50% difference between the normalised brightness levels of the lesioned and non-lesioned striatum.

#### 2.2.7 Peri-stimulus histograms of STN single unit activity

Peri-stimulus histograms (PSTHs) were calculated for the spike trains of each neuron for each frequency with a bin size of 1 ms in relation to the first stimulus of the train (time zero). A z-score was calculated by subtracting the mean of the bin count in the pre-stimulus period of 500 ms before the occurrence of the first stimulus and dividing it by its standard deviation. Inclusion criteria for significant responses were a z-score peak above 2 and at least 6 trials where a spike was fired after at least 4 out of 5 stimuli. In addition, these criteria had to be fulfilled for at least 5 frequencies for the unit to be included. To achieve a significant response during the experiment, neurons were often stimulated with multiple amplitudes and the threshold of response could slightly vary between experimental sessions. Recordings leading to a significant response in the PSTH had a mean stimulation amplitude of 288 +/− 141 μA in controls and 281 +/− 153 μA in 6-OHDA lesioned, both in a range from 100 to 500 μA. Effects at different frequencies were compared within each neuron and across stimuli within each frequency. Please note that the relatively small sample sizes in the final analyses reflect the difficulty of finding STN neurons in vivo*,* holding them for long enough to complete our stimulation protocols and then successfully juxtacellularly labelling and recovering them for post-hoc identification. These neurons were recorded from a wider pool of animals from which STN neurons where recorded, but for several reasons (neuron not responding to stimulation, loosing neurons before all stim protocols were complete and/or labelling etc.) could not be used in this study. Across this larger sample, we found that the neurons we recorded were located across the entire STN. Importantly, however, the neurons used in this study, met all response criteria to the cortical stimulation at multiple frequencies explained above.

#### 2.2.8 Coefficient of Variation (CV) of the first spike following stimulus pulse across trials

For determining the variability of the intervals *(i)* between first spike and stimulus across the consecutive stimuli, we calculated the Coefficient of Variation *(CV)* across trials. The CV can reach a value between 0 and 1 and is defined as *CV* *=* *std(i)/mean(i).* For calculation of the CV for the first stimulus (because here there is no history to define stimulus frequency) all intervals from all frequencies were merged to get a more reliable measure for comparison.

#### 2.2.9 Experimental design and statistical analysis

Due to non-normal distribution of the data we used the Mann-Whitney-*U* Test (MWUT) for pairwise comparison, unless otherwise stated. When more than 2 groups were available we performed a Kruskal-Wallis ANOVA with post hoc Dunn's test unless otherwise stated. To account for within neuron variability, comparisons across consecutive stimuli were performed either by the signed Wilcoxon ranked test (2 comparisons) or the Friedman test (>3 comparisons) with post hoc Tukey-Kramer's tests. All analyses were performed in MATLAB® R2017a (Mathworks). Boxplots for visualization purposes were created in Graphpad Prism (GraphPad Software). Mean values +/− standard deviation and sample sizes are given in the result section. Error bars in all figures show SEM, boxplots show median with the 5–95 percentile.

## 3 Results

The aim of the study was to investigate the selectivity of STN neurons to beta-frequency input and the precise nature of any frequency preference. To address these questions, we utilised two approaches. Firstly, we investigated phase-locking dynamics of STN units to local field potentials and cortical field signals (ECoG and EEG) from 16 PD patients undergoing DBS-surgery. Secondly, we applied short bursts of electrical stimulation to the motor cortex at different frequencies while recording in the STN of 5 anesthetised healthy and 9 anesthetised dopamine-depleted rats.

### 3.1 Entrainment of STN neurons to cortical and local oscillatory input in humans

#### 3.1.1 STN neurons are more strongly phase-locked to beta oscillations than higher power sub-beta oscillations

**Fig. 1:**
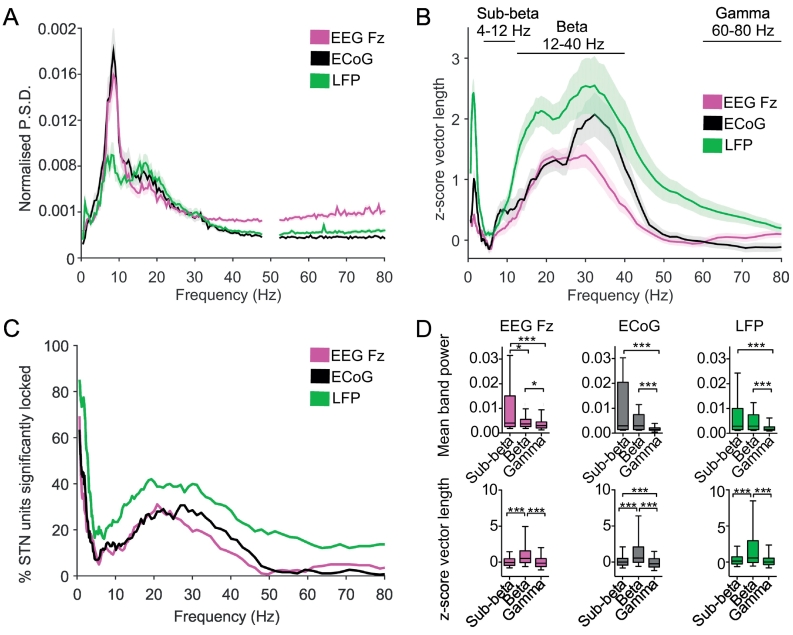
STN units were selectively phase-locked to beta oscillations despite the presence of higher power of oscillatory activity in the sub-beta-frequency range. A, Mean 1/f normalised relative power spectral estimates normalised by the power from 0.5–80 Hz for EEG Fz (n = 142), ECoG (n = 96) and LFP (n = 130). Shaded areas show +/− SEM. Please note that for spectral analysis LFP recordings were averaged per recording depth. B, Phase-locking strength for each of the STN units to EEG Fz (n = 280), ECoG (n = 186) and LFP (n = 248). N-numbers refer to unit-field pairs, in case of LFP recordings phase-locking strength has been averaged for multiple LFP signals per unit prior to group analysis. Note the selectively high phase-locking strength within the beta-frequency range. C, The histogram of the proportion of significant phase-locked units, determined by a significant Rayleigh test, shows a broad peak in the beta-frequency range. D, The mean relative power in sub-beta- (4–12 Hz), beta- (12–40 Hz) and gamma-(60–80 Hz) frequency range (top, same n-numbers as in A) and comparison of the phase-locking strength in the same frequency bands (bottom, same n-numbers as in B). Note the significantly higher phase-locking strength of STN units to beta oscillations despite the higher spectral power for sub-beta oscillations. Significance was tested using a Kruskal-Wallis ANOVA with post hoc Dunn's test. *** *p* < 0.001, * *p* < 0.05. Box plots show the quartile boundaries with whiskers showing the 5–95 percentiles.

Power spectra of LFP/ECoG recordings in patients with Parkinson's disease have peaks in sub-beta, beta and gamma frequencies (Kuhn et al., 2005; Kempf et al., 2009; Zaidel et al., 2010; Shimamoto et al., 2013; West et al., 2016). As LFP recordings are assumed to reflect the summed synaptic input to the STN (Buzsaki et al., 2012), we first investigated whether the power of these subcortical and cortical oscillations is reflected in the activity/level of synchronisation of single STN neurons. While beta oscillations are implicated in PD motor symptoms, patients display higher power oscillations in the sub-beta (4–12 Hz) range (Fz (*n* = 142, F(2,423) = 20.41 *p* = 6.78e-07, post hoc Dunn's *p* < 0.05 between sub-beta/beta and beta/gamma range, for sub-beta/gamma range *p* < 0.001), ECoG (*n* = 96, F(2,285) = 48.37 *p* = 3.14e-11, post hoc Dunn's between sub-beta/beta range *p* = 0.36, between sub-beta/gamma and between beta/gamma range *p* < 0.0001) and LFP (*n* = 130, F(2,387) = 41.76 *p* = 8.54e-10, post hoc Dunn's *p* = 0.88 for comparison sub-beta/beta and *p* < 0.0001 for beta/gamma, sub-beta/gamma), Fig. 1A). In contrast, STN unit phase-locking to beta-frequency oscillations was significantly stronger than at sub-beta frequencies (Fz (*n* = 280, F(2,837) = 88.58 *p* = 5.82e-20, post hoc Dunn's *p* < 0.0001 between sub-beta/beta and beta/gamma range), ECoG (*n* = 186, F(2,555) = 77.16 *p* = 2.89e-17, post hoc Dunn's *p* < 0.0001 for all groups) and LFP (*n* = 248, F(2,741) = 60.95 *p* = 5.81e-14, post hoc Dunn's *p* < 0.0001 between sub-beta/beta and beta/gamma range), Fig. 1B-D). Furthermore, units showed significantly stronger locking to beta-frequency activity than gamma (60–80 Hz). Therefore, in line with previous studies STN spiking was selectively responsive to beta frequencies, despite, higher sub-beta frequency power in the field signals (Moran et al., 2008; Shimamoto et al., 2013; Sharott et al., 2018).

Indeed, across all recordings the peak frequency of phase-locking did not correspond to an obvious peak in the power spectra of the field signals (Fig. 1A vs Fig. 1C). Further analysis showed that some individual patients had matching peaks between field-power and phase-locking, while others did not (Supplementary Fig. 2A-F) and that overall the former had lower frequency peaks than the later (Supplementary Fig. 2G). However, the field power at the peak phase-locking frequency was higher than that of the surrounding frequency bins (Supplementary Fig. 2H), suggesting that there was a relationship between the spectral content of the field signal and the peak frequency of phase locking.

#### 3.1.2 Phase-locking strength of STN neurons to population beta oscillations was dependent on oscillation magnitude

**Fig. 2:**
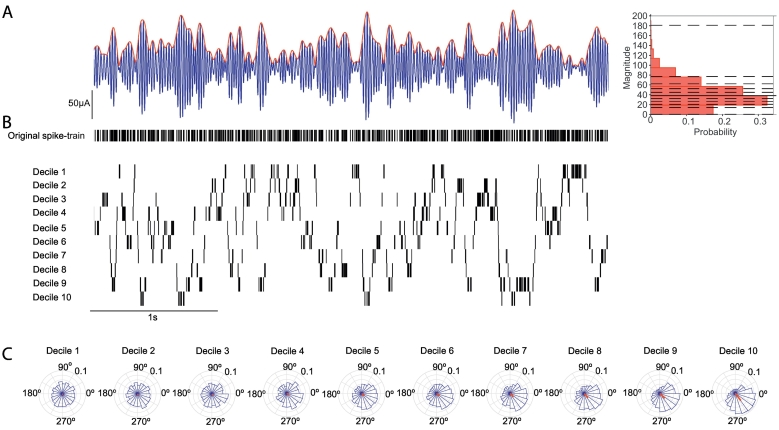
Magnitude dependent phase-locking analysis - schematic illustration. A, Left, Example filtered LFP trace in the beta-frequency range (25–35 Hz) (blue) with instantaneous magnitude of the Hilbert envelope (red). Right, Example distribution of spike-time magnitudes for the example recording shown in A (solid black line: median). Dashed black lines indicate decile divisions. B, Original spike train (top) and spike trains generated by separating spikes occurring within each decile. Data corresponds with the LFP oscillation shown in (A). C, Circular plots show the distribution of spike phases relative to the LFP beta oscillation for each decile. The red line shows the mean phase and vector sum. The vector length becomes longer and the spike phase distribution becomes more peaked with increasing deciles (magnitude of the beta oscillation).

It has been previously shown that, in the motor cortex, the magnitude of population oscillations predicts the engagement of cortical neurons with LFP beta oscillations (Denker et al., 2008). Having established that STN neurons selectively lock to population oscillations in the beta-frequency range, we tested whether the strength of this synchronisation was similarly predicted by the instantaneous magnitude of local and cortical oscillations. Spike-time oscillation magnitudes were divided into deciles (i.e. 1st decile is from 0 to 10% of the spike-time magnitude distribution) (Fig. 2).

**Fig. 3:**
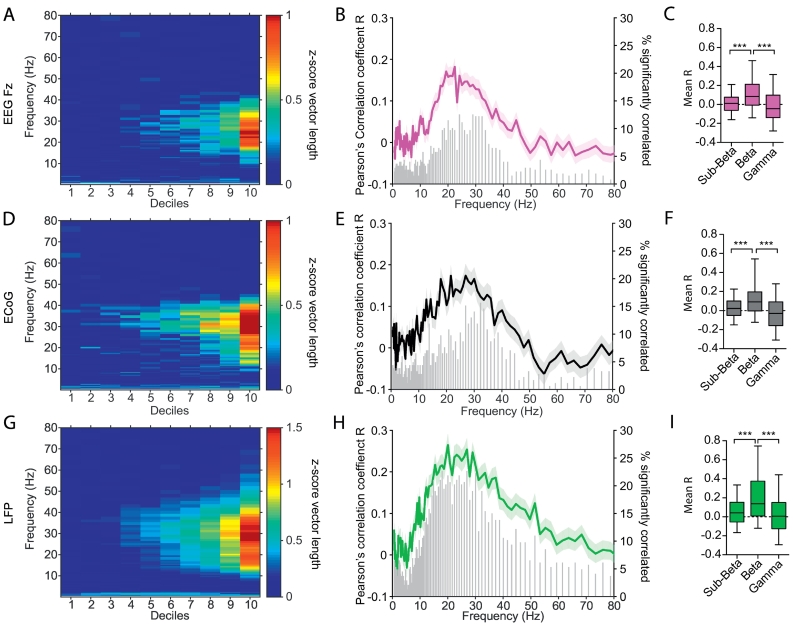
Magnitude dependent increase of STN unit phase-locking strength to population beta oscillations. A, Mean Increase of phase-locking strength of STN units to Fz (*n* = 280 units) in each decile for increasing frequency bins from 0 to 80 Hz. B, For each recording and frequency the Pearson's correlation coefficient (R value) between phase-locking strength and decile number (coloured trace, mean ± SEM, left y-axis) along with the number of significant correlated bins shown in the histogram (grey, right y-axis) were calculated. C, Box plots show the mean correlation coefficient (between decile and z-score vector length) for the sub-beta-(4–12 Hz), beta-(12–40 Hz) and gamma-(60–80 Hz) frequency range (Kruskal-Wallis ANOVA with post hoc Dunn's tests). D-I, Show the same analysis of STN units with ECoG (*n* = 186 units) (D-E) and with LFP (*n* = 248 units) (G-I) recordings. Note the magnitude dependent increase of phase-locking strength in the beta-frequency range for all signals (A,D,G). The highest correlation and amount of bins significantly correlated was likewise highest in the beta-frequency range (B,E,H). The correlation strength was significantly higher in the beta-frequency range in comparison to sub-beta and gamma band power for all network population signals (C,F,I). *** *p* < 0.001, whiskers of box plots show the 5-95th percentile.

We were then able to calculate phase-locking values for spikes occurring within each decile, all with an equal number of spikes due to the magnitude distribution of magnitude values at the spike times. For all investigated network oscillations (LFP, ECoG and EEG Fz), phase-locking strength of STN units increased with the magnitude of beta oscillations, starting around the 6th/7th decile (Fig. 3A,D,G). This correlation between oscillation magnitude and unit phase-locking strength was strongest in the beta-frequency range (Fig. 3B, E, H) (Comparison of the Pearson's correlation coefficient between decile and z-score vector length: Fz (F(2,837) = 85.90 *p* = 2.23e-19), ECoG (F(2,555) = 56.79 *p* = 4.66e-13) and LFP (F(2,741) = 63.13 *p* = 1.96e-14), post hoc Dunn's *p* < 0.0001 for all comparisons). For convenience, units that displayed a significant, positive correlation between phase-locking strength and the magnitude of the coincident LFP, ECoG or EEG signal in the beta-frequency range will be referred to as “magnitude-correlated units” from this point onwards.

#### 3.1.3 Entrainment of STN spiking to beta oscillations increases linearly with the instantaneous magnitude

**Fig. 4:**
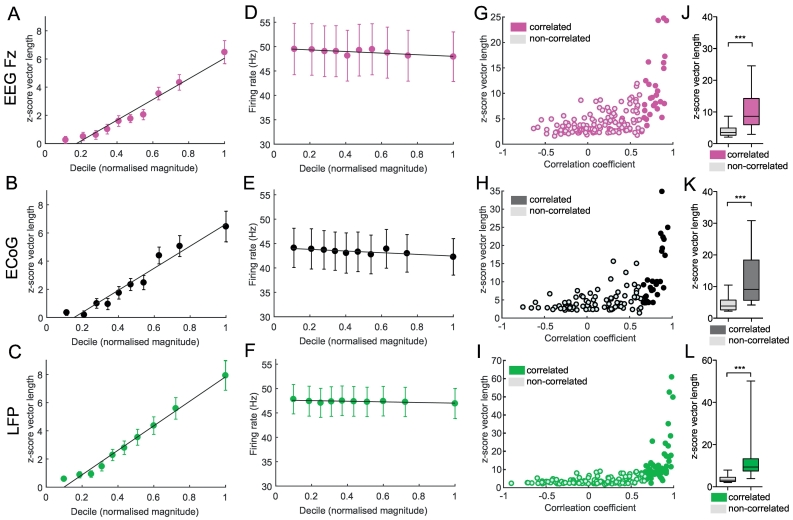
Linear entrainment of STN units to population beta oscillations. A-C, Group average of phase-locking strength (z-score vector length) along the mean normalised magnitude in each decile (x-axis). Error bars show +/− SEM. Group means consist only of magnitude-correlated units for a given signal (Fz: n = 28/154 (A), ECoG: n = 27/109 (B) and LFP: n = 56/172 (C)). The line shows the linear fit of the group average with a significant correlation for all population oscillation signals (Fz: R = 0.96, *p* = 3.35e-05; ECoG: R = 0.98, *p* = 1.23e-06; LFP: R = 1, *p* = 2.67e-09). D-F, Group average of the firing rate +/− SEM in each decile along the mean normalised magnitude in each decile (x-axis) for Fz (D), EcoG (E) and LFP (F). Group averages were limited to consist of the same magnitude-correlated units as in A-C. Note that the majority of units show no correlation between the magnitude deciles and the firing rate (see text) and the mean firing rate remained stable across the deciles. G-I, *Z*-score vector length of each recording plotted against the Pearson‘s correlation coefficients for magnitude-correlation (i.e. the line in A-C now for each unit-field pair). Hollow points represent the non-significantly correlated units and filled dots represent magnitude-correlated units. Note the positive correlation between the correlation coefficients (mean normalised magnitude decile x z-score vector length) and the overall phase-locking strength of the entire recording (Fz: n = 154, Pearson's R = 0.57, *p* *=* 4.71e-15; ECoG: n = 109, Pearson's R = 0.55, *p* *=* 8.67e-10; LFP: n = 172, Pearson's R = 0.51, *p* *=* 6.99e-13). J-L, Statistical comparison (MWUT) of the z-score vector length between non-correlated and magnitude-correlated units revealed a significant higher phase-locking strength for magnitude-correlated units to all population oscillations (Fz (J), EcoG (K) and LFP (L)). This indicates that units that display increasing phase-locking strength with increasing EEG/ECoG/LFP beta oscillation strength (magnitude) over time, have a higher overall phase-locking strength (are more strongly phase-locked during the entire recording). *** *p* < 0.001, box plot whiskers show 5-95th percentile. Additional analyses on the relationship between spike train oscillation power, phase-locking strength and the correlation strength between phase-locking and population oscillation magnitude are shown in Supplementary Fig. 3. Note that for all analyses in this figure the preferred beta-frequency of phase-locking was chosen for each unit-population oscillation pair. A distribution of preferred beta-frequencies is shown in Supplementary Fig. 4 and did not differ between cortical and subcortical signals or oscillatory neuron groups.

The nature of the relationship between magnitude and synchrony could reveal how the activity of individual STN neurons is recruited into population oscillations. Because the magnitude of population oscillations is not normally distributed (Fig. 2A), but increased exponentially over the deciles, we repeated the correlation analysis with the mean normalised magnitude value in each decile (instead of decile number) to get a more accurate estimate of the shape of this relationship. Between 18 and 34% of significantly phase-locked STN units were magnitude-correlated (Fz: *n* = 28/154 (18%), ECoG: *n* = 27/109 (25%) and LFP: *n* = 56/172 (33%)) and correlation coefficients were all close to 1 for these units (Fig. 4A-C, Fz: *R* = 0.96, *p* = 3.35e-05; ECoG: *R* = 0.98, *p* = 1.23e-06; LFP: *R* = 1, *p* = 2.67e-09). To test whether the increase of phase-locking strength was dependent on firing rate, the same analysis was repeated for the firing rate at each decile. The overall firing rate in the majority of cases was not related to the phase-locking strength on a single recording basis (non-correlated firing rate-magnitude units: Fz: *n* = 23/28 (82%), ECoG: *n* = 20/27 (74%) and LFP: *n* = 30/56 (54%)). Where correlations with firing rate were significant, most were negative (Fz: n = 5/28 (18%), ECoG: *n* = 6/27 (22%) and LFP: *n* = 16/56 (29%), with only few significantly positive (Fz: *n* = 0/28 (0%), EcoG: n = 1/27 (4%) and LFP: *n* = 10/56 (18%)). The group average revealed a significant negative correlation for all network population signals (Fz: n = 28, Pearson's R = -0.73, *p* *=* 0.016; ECoG: n = 27, Pearson's R = -0.78, *p* *=* 0.007; LFP: n = 56, Pearson's R = -0.66, *p* *=* 0.03; Fig. 4D-F). Magnitude-correlated units also had the highest overall phase-locking values (Fz: *n* = 154, Pearson's *R* = 0.57, *p* *=* 4.71e-15; ECoG: *n* = 109, Pearson's *R* = 0.55, *p* *=* 8.67e-10; LFP: *n* = 172, Pearson's *R* = 0.51, *p* *=* 6.99e-13; Fig. 4G-I) and their mean phase-locking strength was significantly higher than that of uncorrelated units (MWUT, Fz: *p* *=* 7.16e-10; ECoG: *p* *=* 1.999e-08; LFP: *p* *=* 9.57e-21, Fig. 4J-L).

#### 3.1.4 Entrainment of STN-units to beta oscillations is associated with a higher oscillatory power of STN unit spike trains

Next, we investigated how the phase-locking of single neurons was related to the propensity of those neurons to oscillate at beta frequency. Higher phase-locking strength in the beta-frequency range was associated with a higher oscillatory spike train power at that frequency and neurons with significant oscillatory power had a higher phase-locking strength than neurons that did not show oscillatory activity (Supplementary Fig. 3 A-C). For all field signals we observed a weak but significant correlation between degree of magnitude-correlation and oscillatory power of the spike train (Fz: Pearson's *R* = 0.23, *p* *=* 0.004; ECoG: Pearson's *R* = 0.21, *p* *=* 0.028; LFP: Pearson's *R* = 0.39, *p* *=* 1.20e-07; Supplementary Fig. 3 D,E,F). In line with this result, the mean oscillatory spike train power in the beta frequency of preferred locking was higher for magnitude-correlated units (significant for Fz and LFP), underlining the association between entrainment and oscillatory properties of the unit (comparison of spike train power in preferred beta frequency between non-correlated and magnitude-correlated units (mean normalised magnitude decile x z-score vector length): Fz: (*n* = 126/28), MWUT *p* *=* 0.007; ECoG: (*n* = 82/27), MWUT *p* *=* 0.11; LFP: (*n* = 116/56), MWUT *p* *=* 2.67e-07; Supplementary Fig. 3 D,E,F). Comparing the magnitude-correlation strength of neurons showing oscillatory and non-oscillatory activity revealed significantly higher correlation strengths for neurons showing oscillatory activity (Comparison of mean Pearson's correlation coefficient non-oscillatory vs oscillatory units (MWUT): Fz: (*n* = 106/48), *p* *=* 0.007; ECoG: (*n* = 70/39), *p* *=* 0.002; LFP: (*n* = 125/47), *p* *=* 1.10e-07; Supplementary Fig. 3 D,E,F). These results indicate that oscillatory units have a closer relationship to the strength of ongoing beta oscillations in field signals than non-oscillatory units.

#### 3.1.5 Subsets of STN neurons switch to a synchronised state at different beta oscillation magnitudes

Next we investigated whether neurons that show significant oscillatory activity in their spike trains start phase-locking at lower magnitude values than neurons that did not show oscillatory behaviour. Please note that for reasons of simplification, we will refer to units that fulfil the criteria of significant oscillations (see method sections) in their spike trains as ‘oscillatory units’ and to neurons, that did not as ‘non-oscillatory units’. Supplementary Fig. 1 shows a histogram of beta peak power for oscillatory and non-oscillatory units. While oscillatory units had higher beta peak power than non-oscillatory units, the distribution was not unequivocally bimodal. This could be because non-oscillatory units may also have weak oscillatory activity and/or because the spectral peaks for units were often sharp, making it more difficult to quantify beta power.

**Fig. 5:**
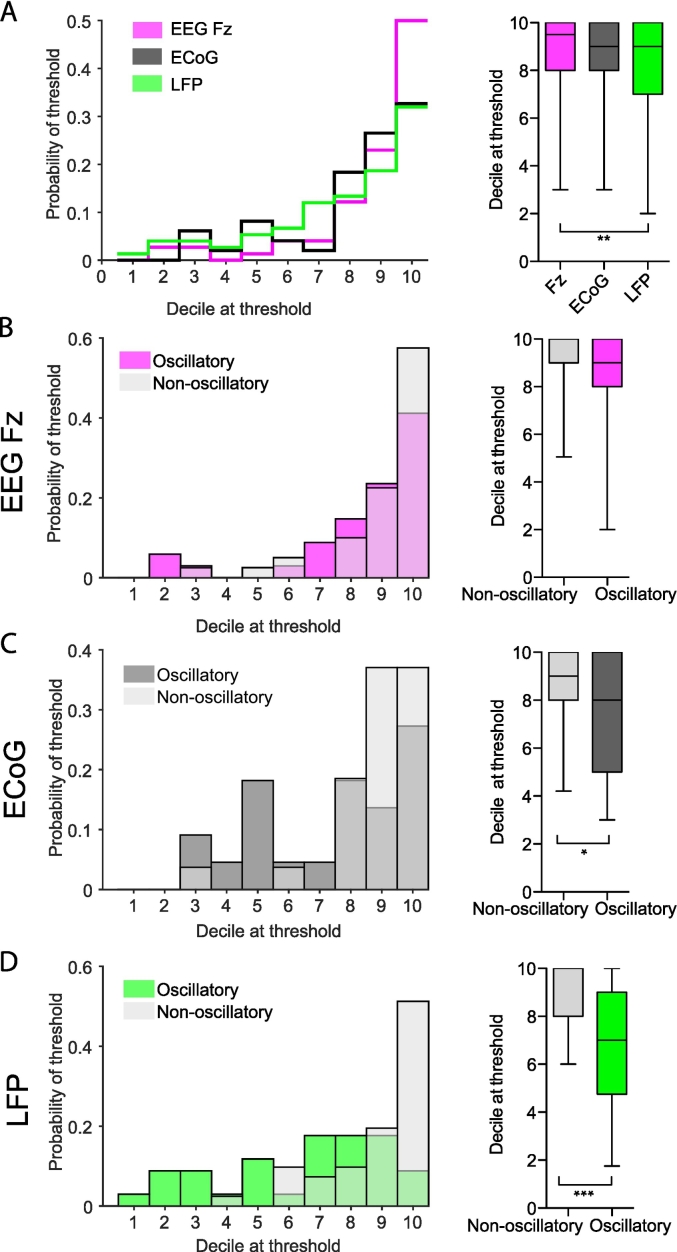
Non-oscillatory STN units require a higher magnitude of population oscillations to become significantly phase-locked. A, Left, distribution of the lowest decile in which each unit becomes significantly locked to the population oscillation. Note that the likelihood increases with decile number. Right, box plots for the distributions on the left. Note the median decile of first locking is the 8th. B-D, Left, distribution of lowest decile in which each unit becomes significantly locked to the population oscillations split into non-oscillatory and oscillatory units for Fz ((B), n = 40 non-oscillatory, n = 34 oscillatory), ECoG ((C), n = 27 non-oscillatory, n = 22 oscillatory) and LFP ((D), n = 41 non-oscillatory, n = 34 oscillatory). Intermediate colours indicate the overlap of the histogram between oscillatory and non-oscillatory units. Note that the threshold for non-oscillatory units to have significant phase locking is often the 10th decile, whereas the threshold for many oscillatory units is lower. Right, boxplots of the distributions on the left. Note the decile of first significant locking is higher for non-oscillatory units for all population signals, with a significant difference for ECoG and LFP. Pairwise comparisons were performed with MWUT, *** *p* < 0.001, *p < 0.05, whiskers of boxplots show the 5-95th percentile.

On average, significant phase-locking started around the 8th magnitude decile and was lower for the LFP than Fz (Mean decile at threshold of significant phase-locking: Fz: 8.78 +/− 1.91 (*n* = 74); ECoG: 8.18 +/− 2.10 (*n* = 49); LFP: 7.77 +/− 2.42 (*n* = 75); Kruskal-Wallis ANOVA F(2,192) = 12.15 *p* = 0.01, post hoc Dunn's test *p* = 0.01 between Fz and LFP; Fig. 5A). Non-oscillatory units had a higher phase-locking threshold than oscillatory units, often only showing significant phase-locking at the highest decile (Fz: non-oscillatory (*n* = 40) 9.08 +/− 1.58, oscillatory (*n* = 34) 8.41 +/− 2.22, MWUT *p* *=* 0.11; ECoG: non-oscillatory (*n* = 27) 8.85 +/− 1.51, oscillatory (*n* = 22) 7.36 +/− 2.44, *p* *=* 0.036; LFP: non-oscillatory (*n* = 41) 8.85 +/− 1.59, oscillatory (n = 34) 6.47 +/− 2.65, *p* *=* 1.18e-05; Fig. 5B-D). Note that only neurons with a detectable threshold were included. The preferred frequency of phase-locking did not differ between non-oscillatory and oscillatory units (distribution shown in Supplementary Fig. 4). While non-oscillatory STN units did not show strong correlations with beta magnitude, a subpopulation of oscillatory units was more likely to be strongly magnitude-correlated as well as significantly phase-locked at lower magnitude values (Fig. 5 and Supplementary Fig. 3). Therefore, subsets of STN neurons displayed different phase-locking behaviours. Non-oscillatory units tended to exhibit a discrete state change, only phase-locking to beta oscillations at the highest magnitudes or not at all, while oscillatory units could display a positive linear correlation across the entire magnitude range, making it possible to predict their behaviour by LFP and cortical beta activity.

#### 3.1.6 Enhanced phase-locking strength of STN-neurons during beta bursts

**Fig. 6:**
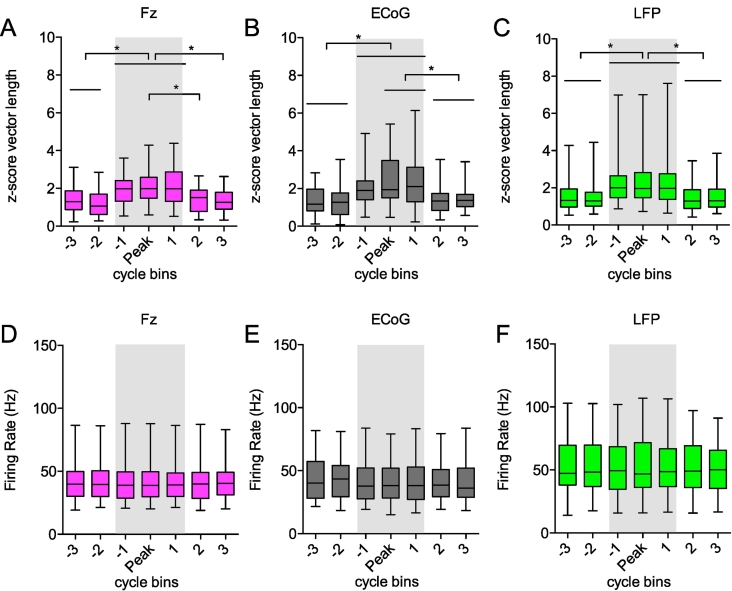
Entrainment of STN-units during periods of elevated beta power (beta bursts). A-C, Phase-locking analysis of STN-units before, during and after episodes of elevated beta power. Beta bursts were defined as periods of elevated beta power that exceeded the 75th percentile threshold of the magnitude for EEG Fz (A, pink, *n* = 49), ECoG (B, grey, *n* = 38) and LFP (C, green, *n* = 64). For each threshold crossing of more than 3 cycles the two cycles before that of the rising threshold crossing, the three cycles around the peak magnitude and the two cycles after the falling threshold crossing are depicted. Only STN units/multi-units that showed a positive correlation of phase-locking strength with the magnitude of the oscillation are included. Note the significantly higher phase-locking strength during episodes of elevated power (inside the grey shaded area). * indicate significant *p*-values of Dunn's post hoc comparison. Units that showed no correlation of phase-locking strength with the magnitude of the oscillation are shown in Supplementary Fig. 5 and showed no increase of phase-locking strength during beta bursts. D-E, Mean firing rate in corresponding cycle bins showed in A-C for STN units during and around episodes of elevated beta power (beta bursts) in EEG Fz (D), ECoG (E) and LFP (F). A Kruskal-Wallis ANOVA reveals no significant difference of the firing rate within and outside a beta burst.

Having shown that the magnitude of oscillations predicts the entrainment of STN-units, we next evaluated the temporal evolution of such entrainment by investigating this relationship over the timecourse of high amplitude “bursts”, which are associated with hypokinetic symptoms and can be used as a marker for therapeutic strategies (Tinkhauser et al., 2017b; Tinkhauser et al., 2017a). Phase-locked STN units were divided in two groups, those that were magnitude-correlated and those that were not magnitude-correlated but were significantly phase-locked across the whole recording (phase-locked only). For magnitude-correlated units, there were significant differences in the phase-locking strength before, during, and after identified bursts (Fz: F(6,336) = 50.94, *p* = 3.04e-09; ECoG: F(6,257) = 39.38, *p* = 6.02e-07; LFP: F(6,434) = 53.31 *p* = 1.02e-09; Kruskal-Wallis ANOVA, post hoc Dunn's test significant values shown in Fig. 6A). In contrast, the firing rate was not significantly different before, during or after identified beta burst (Fz: F(6,336) = 0.28 *p* = 1; ECoG: F(6,259) = 0.89 *p* = 0.99; LFP: F(6,441) = 0.36 *p* = 1).

In contrast, STN units that were not magnitude-correlated showed no increase of phase-locking during beta bursts (Fz: F(6,666) = 8.06 *p* = 0.20; ECoG: F(6,461) = 4.90 *p* = 0.56; LFP: F(6,623) = 3.69 *p* = 0.718) and no firing rate changes (Fz: F(6,686) = 0.15 *p* = 0.99; ECoG: F(6,476) = 0.36 *p* = 0.99; LFP: F(6,651) = 0.41 *p* = 0.99) (Supplementary Fig. 5). These results indicate, that phase-locking is high during episodes of elevated beta power for a subset of significantly phase-locked STN-neurons (Fz: n = 49/148 (31%); ECoG: n = 38/107 (36%); LFP: n = 64/158 (41%)).

### 3.2 Entrainment of identified STN neurons to beta oscillatory input in rats

#### 3.2.1 Cortical stimulation at different frequencies with simultaneous juxtacellular recordings in the STN of 6-OHDA hemi-lesioned rats and controls

**Fig. 7:**
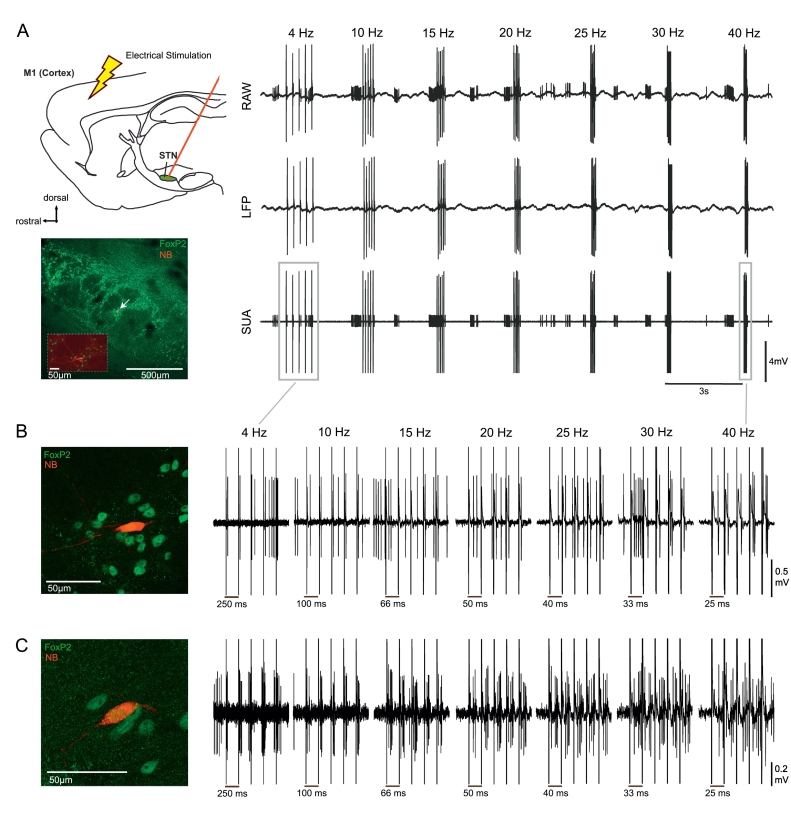
Recording setup and stimulation protocol for rat experiments. A, Left top, schematic illustration of the recording setup. Recordings were performed with a glass pipette located in the STN and simultaneous electrical stimulation of the motor cortex. Left bottom, a Neurobiotin (NB) labelled neuron located in the STN, which was delineated using forkhead box protein P2 (FoxP2) immunoreactivity. A higher magnification image of the labelled neuron is inset. Right, raw recording tracesduring one block of stimulation within the 7 frequencies 4,10,15,20,25,30 and 40 Hz. For each frequency a burst of 5 consecutive stimuli was applied. This protocol was repeated 12 times. Top row, the raw signal was low-pass filtered at 2000 Hz and used to extract the LFP. Middle row, the LFP signal after the removal of spikes as described. Bottom row, single unit activity was extracted following band-pass filtering between 300 and 5000 Hz. B, The same Foxp2-expressing neuron shown in A, which was recorded in a 6-OHDA lesioned rat. Right, raw data recorded from the labelled neuron. The period around each burst of stimuli for each individual frequency has been magnified. Note the time scale of the plots differs. In this example, 4 Hz stimulation results in the timing the evoked spikes having high variance over the course of the stimuli, which is reduced at higher frequencies. C, Left, a FoxP2-expressing labelled neuron from a healthy rat. Right, the corresponding raw data traces from the labelled neuron.

The analysis of STN neurons recorded in PD patients described above suggests that they are selectively sensitive to changes in the magnitude of ongoing beta oscillations in cortex. However, these analyses are dependent on natural fluctuations in the magnitude of ongoing oscillations. Moreover, we cannot determine whether this beta-selectivity is a function of the parkinsonian brain, as such data cannot be recorded from control subjects. To address these issues experiments were carried out in anaesthetised rats, where we could manipulate the frequency of cortical input to STN neurons with a fixed magnitude in control and dopamine depleted states (Fig. 7). Approximately half of the recorded STN neurons showed a significant response (z-score > 2, for details see methods) in the PSTH (8/13 (62%) control, 8/17 (47%) 6-OHDA lesioned rats) to 4–40 Hz cortical stimulation (M1). Example PSTHs for a neuron from a control and a 6-OHDA lesioned rats demonstrated the multiphasic response profile described previously (Magill et al., 2004) (Fig. 9). Note that the most prominent peaks are preserved at different stimulation frequencies.

#### 3.2.2 STN-neurons in the cortico-pallidal-subthalamic network show frequency selective entrainment to cortical stimulation in healthy and parkinsonian rats

**Fig. 8:**
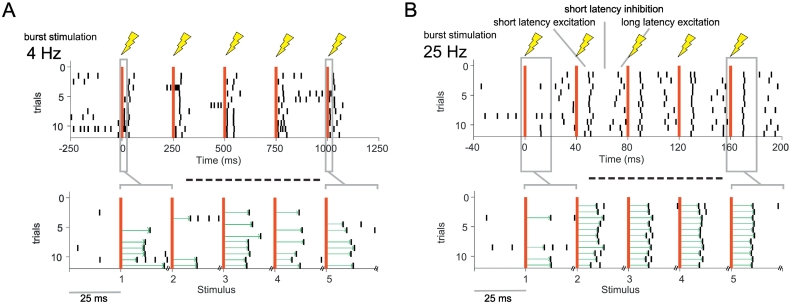
Schematic illustration of spike time variability analysis. A, Top, Example stimulation of a stimulus train of 5 consecutive stimuli at 4 Hz defined by the inter-stimulus interval of 250 ms. Red lines indicate the timing of the stimulus. The burst oscillation frequency is only defined after the second pulse. Bottom, to examine spike time variability of the first spike, we show only the first 25 ms after each stimulus. Red lines mark the stimuli but the x-axis in not continuous, showing only the first 25 ms post-stimulation. Green arrows indicate the interval between stimulus and the first spike. B, Top, as in A but for a stimulus train at 25 Hz/inter-stimulus interval of 40 ms. Bottom, as in A, only first 25 ms after each stimulus is shown. Note that the alignment of the first spike is increasing after the second stimulus. Using this presentation format, the alignment of the first spike after *each* stimulus can be visualised easily across trials, irrespective of the stimulation frequency.

Heterogeneity in the firing rate and pattern of STN neurons, either occurring naturally in the healthy brain or as a result of dopamine depletion, resulted in overtly different responses to stimulation trains between neurons (Fig. 7). We next designed an analytical approach to try and capture the core response properties of STN neurons to different stimulation frequencies beyond this heterogeneity. To measure neuronal synchronisation to a given stimulus train at a certain frequency, we calculated the Coefficient of Variation (CV) of the first spike occurring after each stimulus pulse. The CV measures the spike time variability of the first spike after each stimulus illustrated by green arrows in Fig. 8 and allows to investigate changes in the consistency of response latency during stimulus bursts at different frequencies.

**Fig. 9:**
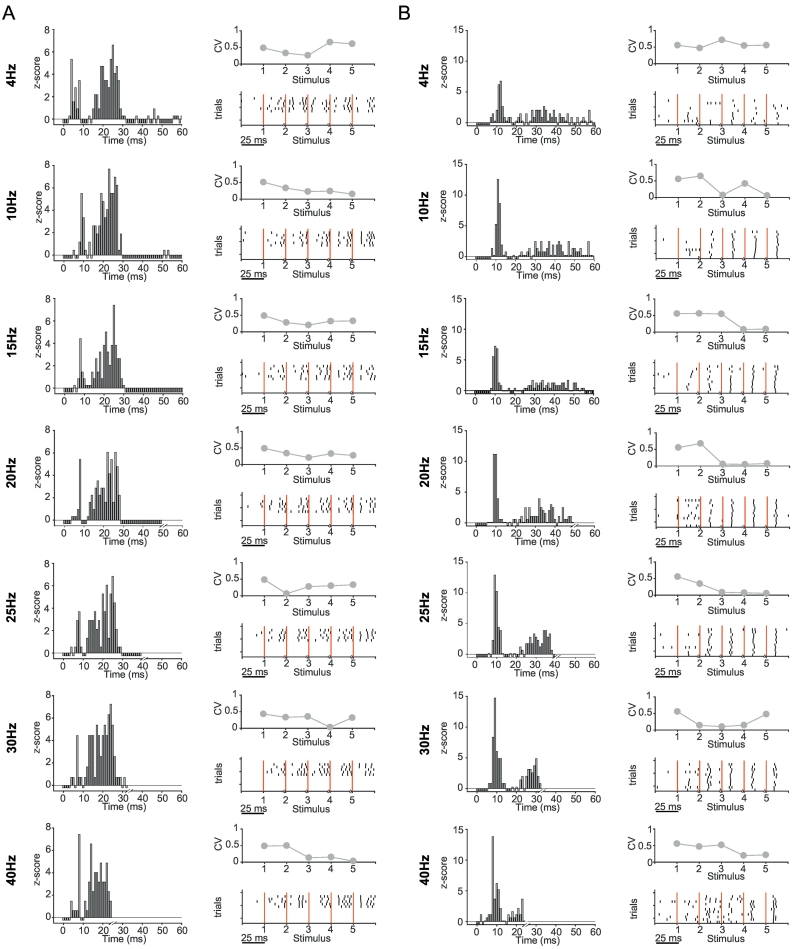
Example responses of identified STN-neurons to cortical stimulation at different frequencies. A,B, Each column shows the response of an STN neuron from a control rat (A) and 6-OHDA hemi-lesioned rat (B) to alternating stimulation in the frequencies 4, 10, 15, 20, 25, 30 and 40 Hz (rows) with 5 consecutive stimuli during one recording session. Left, Normalised PSTH (z-score, based on 500 ms of pre-stimulus period) to the stimulation at time 0. All consecutive stimuli were merged for the calculation of the PSTH. Note that the observation window decreases with the stimulation cycle period. Right bottom, rasterplot of all available trials for the STN neuron. The red line indicates the stimulus timing, with in total 5 consecutive stimuli per trial. Each line represents 1 trial in the recording session. Note that the time scale was matched to be equivalent for all frequencies and therefore for every frequency only 25 ms after each stimulus are shown and the x-axis is non-continuous, as demonstrated in Fig. 8. Right Top, Coefficient of Variation (CV) calculated of the first spike after the stimulus calculated across stimulus trials was used to quantify amount of synchronisation to each stimuli in the train. Note the decreasing Coefficient of Variation (CV) in the frequencies 10–25 Hz in both examples, indicating an increasing synchronisation as the stimulus train progresses.

The rasterplots in Fig. 9 show the increasing consistency in the timing of the first evoked spike at 10, 15, 20 and 25 Hz for two example neurons (Fig. 9), suggesting increased synchronisation over consecutive pulses.

**Fig. 10:**
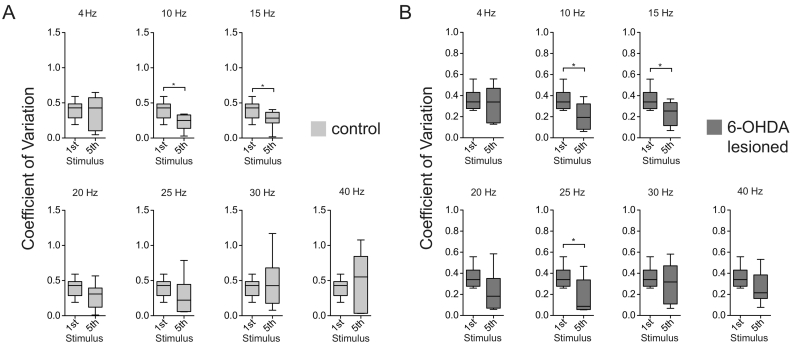
Frequency selective entrainment of identified STN-neurons to cortical stimulation in control and 6-OHDA lesioned rats. A,B, Group analysis for the Coefficient of Variation (CV) of the first spike after each stimulus across trials. The CV is used as a measurement of spike synchronisation to the stimulus. Data from all frequencies was used to calculate the CV of the first stimulus. Comparison of the Coefficient of Variation (CV) between 1st and 5th stimulus (because the likelihood to detect a difference will increase with the course of stimuli) for control (n = 8 STN neurons) (A) and 6-OHDA lesioned (n = 8 STN neurons) (B) rats across frequencies 4, 10, 15, 20, 25, 30 and 40 Hz. Box plots showing 5–95 percentile. * indicates *p* < 0.05 of the signed Wilcoxon rank test. Note the significant difference for 10 and 15 Hz in both groups, and the slightly more pronounced and significant difference at 25 Hz in 6-OHDA lesioned rats. Overall both groups show a trend of a lower CV at the 5th stimulus in the frequencies between 10 and 25 Hz, indicating that STN neurons are more likely entrained by cortical beta frequency input in both groups.

When comparing the 1st and 5th stimulus, in both control and lesion groups the CV showed a tendency to decrease after the 5th stimulus selectively in the frequencies 10–25 Hz (Fig. 10). A significant decrease in CV following the 1st and 5th stimulus occurred for 10 and 15 Hz in controls (*n* = 8, 10 Hz: *p* = 0.008;15 Hz: *p* = 0.039) and 6-OHDA lesioned animals (n = 8, 10 Hz: *p* = 0.039; 15 Hz: *p* = 0.016). In addition, 6-OHDA lesioned animals showed a significant difference at 25 Hz (n = 8, *p* = 0.023), although there was a similar trend in controls. The tendency of STN neurons to be entrained by beta-frequency cortical input was therefore not necessarily a specific property of dopamine depleted animals.

#### 3.2.3 Increased synchronisation to cortical stimulation at beta frequencies is not accompanied by an increase in firing probability

**Fig. 11:**
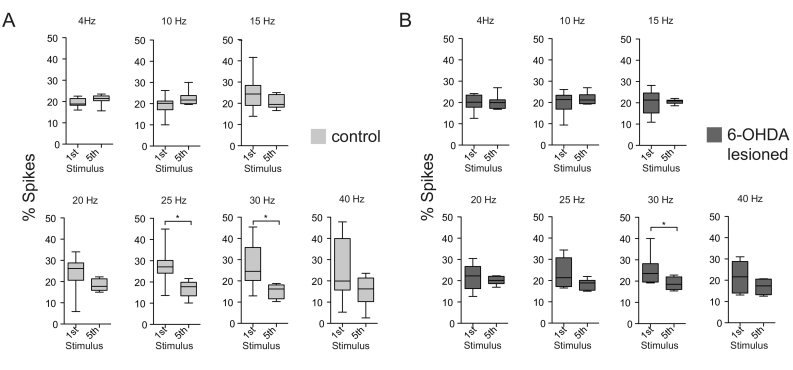
Trend of decrease in firing probability of STN neurons over the course of cortical stimulation at beta frequencies. Firing probability after each stimulus expressed in % spikes after each stimulus with respect to all spikes during the burst of stimuli. Each subplot shows the firing probability in the first 30 ms after the first stimulus in comparison to the 5th stimulus for control (n = 8 STN neurons) (A) and 6-OHDA lesioned (n = 8 STN neurons) (B) rats across frequencies 4, 10, 15, 20, 25, 30 and 40 Hz. Box plots showing 5–95 percentile. * indicates p < 0.05 of the signed Wilcoxon rank test. This indicates that selectively in the frequencies 25 and 30 Hz the firing probability gets suppressed. Overall both groups show a trend of a lower firing probability at the 5th stimulus in the frequencies 20–40 Hz, indicating that STN neurons are less likely to discharge at stimulation at higher frequencies. Together with the CV-Analysis this suggests, that increase in spike time sychronisation is associated with a decrease – or at least lack of increase- in the beta frequencies.

Having shown that the spiking of STN neurons is more precisely timed by beta-frequency input, we examined whether this was associated with changes in firing rate. Fig. 11 shows a comparison of the firing probability between the 1st and 5th stimulus for each frequency. For controls, there was a significant decrease in firing probability after the 5th stimulus for 25 and 30 Hz (n = 8, 25 Hz: *p* = 0.02; 30 Hz: *p* = 0.02). All other frequencies did not show any significant change in firing probability across stimuli (n = 8, 4 Hz: *p* = 0.22; 10 Hz: *p* = 0.38; 15 Hz: *p* = 0.11; 20 Hz: *p* = 0.15; 40 Hz: *p* = 0.22, Wilcoxon rank test) (Fig. 11A). The 6-OHDA lesioned animals also showed a significant decrease in firing probability at 30 Hz (n = 8, 30 Hz: *p* = 0.04), while the other frequencies did not show significant differences across stimuli (n = 8, 4 Hz: *p* = 0.69; 10 Hz: *p* = 0.95; 15 Hz: *p* = 1; 20 Hz: *p* = 0.58; 25 Hz: *p* = 0.11; 40 Hz: *p* = 0.25) (Fig. 11B).

In summary, these changes suggest that synchronisation at beta-frequency input is accompanied by a constant or decreased firing probability in line with results from human data.

## 4 Discussion

Abnormally sustained phase-locking of STN neurons and other basal ganglia populations to beta-frequency population oscillations in PD is thought to limit information coding space, ultimately leading to akinetic/rigid symptoms (Engel and Fries, 2010; Brittain et al., 2014; Khanna and Carmena, 2015; Sharott et al., 2018). It remains unclear, however, why these networks resonate at beta frequency and whether this frequency selectivity is engrained into the system, or is purely pathological. In this study, we show that (1) STN neurons are phase-locked selectively to cortical and subcortical beta frequency oscillations in a magnitude-dependent manner and (2) that STN neurons are more likely to be entrained to 10–25 Hz stimulation in comparison to low (4 Hz) and higher frequency (30-40 Hz) cortical stimulation in both healthy and dopamine-depleted rats. These results suggest that STN neurons can be preferentially driven by beta-frequency cortical input that is unmasked under parkinsonian conditions, and that this preference may be due to the connectivity of the microcircuit in which they are embedded.

#### 4.1.1 Frequency-selective and magnitude-dependent entrainment of STN neurons to population oscillations in PD patients

Phase-locking of single neurons to population signals provides an important metric in PD pathophysiology, as it indicates that individual neurons have become engaged with activities that are synchronised across the network (Mallet et al., 2008a; Mallet et al., 2008b; Cagnan et al., 2019). It was recently demonstrated that the scale of such synchronisation correlates with motor impairment (Sharott et al., 2018). While STN neurons lock to frequencies across a wide beta range (Shimamoto et al., 2013; Sharott et al., 2018), we found that they rarely do so with other frequencies. The relatively low incidence of locking to sub-beta oscillations, which have higher or equivalent power in the cortex and STN LFP, indicates that the selectivity of STN units to beta frequencies is not a trivial result of increased power, but rather reflects an intrinsic property of the neurons or the network in which they are embedded.

We found that a subpopulation of neurons had a positive linear relationship, between instantaneous beta amplitude and phase-locking. Importantly, these magnitude-correlated neurons were most likely to oscillate at beta-frequency, and thus be of particular relevance to akinetic/rigid disease symptoms (Sharott et al., 2014). The full dynamic range of LFP amplitude, therefore, gives the most information about the neurons that are most strongly related to disease pathology. Another subset of neurons became significantly phase-locked at the highest amplitudes, indicating they are less sensitive to the temporal dynamics of oscillatory input. The remaining phase-locked neurons had no observable relationship with instantaneous amplitude. Here, we cannot rule out that we were recording a suboptimal population signal for those units, for example an unconnected cortical area or too distant LFP recording position. However, as the neurons were phase-locked to that signal over the entire recording*,* this result suggests that some neurons are genuinely only weakly coupled to population oscillations and their temporal dynamics.

Our results are in line with and complementary to the study by Moran and colleagues, who were able to categorise individual STN units based on the strength of their phase-locking to the tremor and beta oscillations in the background population (Moran et al., 2008). As in that study, the majority of STN neurons here were uncoupled to local oscillations and their spike trains were non-oscillatory, while around 20–40% were significantly phase-locked. We extend these findings to show that within this oscillatory group, individual units display a range of synchronisation that varies in relation to the dynamics of the population signals. Out of all significantly phase-locked neurons, 20–30% are magnitude-correlated, indicating a further functional significant class of STN neurons that can engage with cortical oscillation strength. These neurons were more likely to display oscillatory spiking, indicating that neurons expressing significant oscillatory activity in their spike trains are most sensitive to the level of oscillation present across the network.

It is also important to consider the relationship between the magnitude of the oscillation and the signal-to-noise of the phase estimate from the same signal. As a general rule, when the magnitude is high the phase estimate is more accurate. Thus, when there is a significant phase-locking relationship between a field and a given unit, it will be more readily detected when the magnitude is high. However, the direction of causality between these processes is somewhat arbitrary, as both a stable phase estimate and high oscillatory power require synchronised synaptic input to/action potential firing of the underlying population. Our findings demonstrate that in the STN, increased power and/or phase stability do not necessitate that the spike trains of all local neurons are entrained. In line with our recent study of entrainment of different basal ganglia cell-types to beta oscillations in 6-OHDA lesioned rodents (Cagnan et al., 2019), the findings here demonstrate that either ECoG/LFP beta magnitude or phase consistency can be used as proxy signals to predict consistent patterns of spiking produced by a sub-population of neurons that are sensitive to entrainment by beta oscillations. While alterations in spiking will ultimately dictate function and dysfunction, the greater accessibility of ECoG/LFPs means they are more commonly used to interpret the physiological and pathological role of beta oscillations. By providing a quantitative link between these processes, the findings presented here have the potential to provide more accurate interpretations as to how these changes in ECoG/LFP activity are likely to be reflected at the single unit level.

#### 4.1.2 Temporal dynamics of neuronal synchronisation in the STN

The instantaneous amplitude of beta oscillations has gained increasing importance given the recent focus on the transient nature of beta oscillations in sensorimotor circuits, often referred to as “beta bursts,” in both health (Feingold et al., 2015; Sherman et al., 2016; Khanna and Carmena, 2017; Shin et al., 2017) and disease (Tinkhauser et al., 2017b; Tinkhauser et al., 2017a; Lofredi et al., 2018; Tinkhauser et al., 2018; Torrecillos et al., 2018). In addition, beta bursts are an effective biomarker for “adaptive” DBS in PD patients (Little et al., 2013). However, none of these studies have demonstrated how such events influence underlying processing by individual neurons. In particular, it is not clear whether thresholding the ongoing LFP or ECoG/EEG power represents a point in a continuum or a step change in the engagement of the underlying neurons with an incoming oscillatory input and/or local synchronisation. This relationship between the LFP and spiking is crucial, as it will ultimately dictate the impact of the oscillation on coding within STN and in downstream structures.

Our analysis of the relationship between LFP/EEG amplitude and phase-locking suggests that, in the STN, commonly used thresholds appear well designed to capture maximum engagement units to population oscillations. These analyses also show, however, that such thresholds may exclude valuable information occurring at lower amplitudes. This finding has important implications for the design of closed-loop stimulation for the treatment of PD. A high threshold may well be optimal for adaptive DBS, where the aim is to disrupt periods of maximum synchronisation as they occur. For other approaches, it could be possible to utilise the lower amplitude information. Several authors have postulated that phase-dependent stimulation could be an effective therapeutic strategy for treating motor symptoms (Rosin et al., 2011; Azodi-Avval and Gharabaghi, 2015; Holt et al., 2016; Meidahl et al., 2017) and we have recently demonstrated the potential efficacy of this approach (Holt et al., 2019; McNamara et al., 2020).

#### 4.1.3 Beta frequency cortical input selectively reduces spike-time variance

Using cortical stimulation in rodents allowed us to provide a controlled oscillatory input of equal amplitude, but variable frequency, to identified STN neurons in both healthy and dopamine-depleted states. Cortical stimulation obviously differs significantly to physiological oscillations generated by a network. Nevertheless, using stimulation enabled us to make a fairer comparison across frequencies than in the patient data, where one frequency tends to dominate in each patient (Zaidel et al., 2010; Sharott et al., 2018). The frequency range used for stimulation here (4–40 Hz) contained frequencies considerably outside the beta range of anaesthetised 6-OHDA lesioned rat, which is consistently around 20 Hz, and rarely goes above 25 Hz (Mallet et al., 2008a; Mallet et al., 2008b; Sharott et al., 2017). Only stimulation at 10–15 Hz, and in case of 6-OHDA lesioned rats at 25 Hz, decreased the variability of the timing of evoked spikes, broadly consistent with a higher fidelity of locking of the STN neurons to beta input in the patient data.

While there were several points of consistency between the human and rodent experiments, we also found that STN neurons had a significantly lower tendency to lock to sub-beta frequency cortical and LFP oscillations in PD patients, whereas cortical stimulation around this frequency significantly reduced spike-time variability at these frequencies. One possible explanation for this confound is that the interval for 10 Hz stimulation is close to the commonly used start of the low beta range (~ 12 Hz). Driving the oscillation electrically may be enough to trigger entrainment that would usually be seen at slightly higher frequencies. Indeed, sub-beta and low-beta power in STN LFPs is modulated to a similar degree by dopamine replacement in PD patients (Priori et al., 2004), albeit with a less strong effect for sub-beta. However, it is possible that the artificial nature of the stimulus and/or species difference may not model the parkinsonian oscillations in all respects. The importance of the precise frequency of synchronisation across the wide beta band is still an intense matter of debate in the field with considerable variability within PD patients, and between PD patients and some animal models (Priori et al., 2004; Zaidel et al., 2010; Quiroga-Varela et al., 2013; Oswal et al., 2016; Sharott et al., 2018). The frequency of oscillatory activity in the MPTP primates (8–15 Hz) is considerably lower on average than in patients, but has nevertheless given crucial insights into PD pathophysiology. The results of the stimulation experiments presented here demonstrate a “band-pass” frequency selectivity in cortico-subthalamic entrainment, which could differ in frequency range between species and recording conditions. Interestingly, only the 6-OHDA lesioned group had a reduction in spike-time variability in the higher beta range. Increased sensitivity to this frequency would be consistent with the frequency of spontaneous oscillations in this model, with the typical beta peak occurring at 20–25 Hz during general anaesthesia (Mallet et al., 2008a; Mallet et al., 2008b). This result suggests that even when using strong, artificial entrainment of the cortico-subthalamic pathways, dopamine depletion changes the nature of frequency response of individual neurons.

Given that we observed a stable number of spikes (lower beta frequencies [10–20 Hz]) or a concurrent reduction in the number of spikes fired during higher beta stimulation (25–30 Hz), a trivial explanation for frequency selectivity is that intrinsic properties of STN neurons do not allow spiking to follow higher frequencies, reducing variance in spike timing. Several lines of evidence suggest that this is not the case. EPSPs in STN neurons reduce, rather than increase, the firing threshold immediately after a spike is fired (Farries et al., 2010) and can follow higher frequency inputs than used here (Bevan and Wilson, 1999; Do and Bean, 2003). Indeed, STN neurons do not display any specific resonance in their intrinsic properties (Farries et al., 2010). Together with our finding of constant firing rates during beta bursts in humans, we did not find that beta frequency oscillations were associated with firing rate increases under physiological or experimental conditions.

#### 4.1.4 Implications for involvement of specific basal ganglia pathways in beta-frequency selectivity

While the in vivo recordings used here did not allow us to examine intrinsic dynamics directly, they have the advantage allowing the measurement of the STN neuron response in the intact network. Using in vitro recordings, Bevan and colleagues have demonstrated that the simultaneous arrival of IPSPs is necessary for oscillatory EPSPs to entrain spiking at the input frequency in STN neurons (Baufreton et al., 2005). Based on this finding, they proposed that the simultaneous IPSPs would result from the polysynaptic activation of the reciprocal pathway with GPe (Baufreton et al., 2005; Baufreton et al., 2009). Based on characterisation of the multiphasic response of STN neurons to single pulses of cortical stimulation (Nambu et al., 2000; Magill et al., 2004; Farries et al., 2010; Janssen et al., 2017), this reciprocal inhibition would be expected to arrive at around 10–15 ms after a cortical stimulus. This would be considerably earlier than the following pulse would arrive during a beta frequency train (40–100 ms). Rather, this time window (40–100 ms) is when the indirect pathway input has been hypothesised to arrive in these cortical stimulation studies. Therefore, we also hypothesize that a coincident inhibitory drive is necessary to entrain STN neurons to cortical input, but that is mediated by the indirect pathway.

This interpretation concurs with recent work suggesting that the hyperexcitability of indirect pathway spiny projection neurons following dopamine depletion could allow entrainment of GP by cortical input, which would usually be dampened in the presence of dopamine (Sharott et al., 2017; Cagnan et al., 2019). Cortical stimulation could achieve similar entrainment to that seen in PD by artificially driving striatal projection neurons that would otherwise be quiescent (Mallet et al., 2006; Sharott et al., 2017). Thus, we propose that the results in both control and lesion animals here are mimicking what happens in the parkinsonian brain, where the indirect pathway is abnormally recruited by cortical input. If this is the case, novel therapeutic strategies that prevent rhythmic indirect pathway input to STN could tip the balance back towards intrinsic firing and reduce motor impairment.

## 5 Conclusions

We have demonstrated that the pattern of spiking of STN neurons is influenced more by cortical beta-frequency input, defined using spontaneous fluctuations in the amplitude of EEG and ECoG in PD patients or using cortical stimulation in rodents, than other frequencies. Because this beta-selectivity was present in the intact and dopamine-depleted brain, we hypothesize that beta-frequency input provides an optimal temporal time window to entrain STN-neurons. These results support the idea of a beta-frequency specific resonance of STN neurons, and/or the network in which they are embedded. Further studies are needed to disentangle the contribution of different pathways and intrinsic properties contributing to this feature of the STN.

## Declaration of Competing Interest

C.K.E.M has served as a medico-scientific consultant to Abbott/St. Jude Medical and Alpha Omega. A.G. declares occasional travel reimbursement from Medtronic.

## Appendix A Supplementary data

Supplementary material Image 1

## References

[bb0005] Azodi-Avval R., Gharabaghi A. (2015). Phase-dependent modulation as a novel approach for therapeutic brain stimulation. Front. Comput. Neurosci..

[bb0010] Baufreton J., Atherton J.F., Surmeier D.J., Bevan M.D. (2005). Enhancement of excitatory synaptic integration by GABAergic inhibition in the subthalamic nucleus. J. Neurosci..

[bb0015] Baufreton J., Kirkham E., Atherton J.F., Menard A., Magill P.J., Bolam J.P., Bevan M.D. (2009). Sparse but selective and potent synaptic transmission from the globus pallidus to the subthalamic nucleus. J. Neurophysiol..

[bb0020] Berens P. (2009). CircStat: a MATLAB toolbox for circular statistics. J. Stat. Softw..

[bb0025] Bergman H., Wichmann T., Karmon B., DeLong M.R. (1994). The primate subthalamic nucleus. II. Neuronal activity in the MPTP model of parkinsonism. J. Neurophysiol..

[bb0030] Bevan M.D., Wilson C.J. (1999). Mechanisms underlying spontaneous oscillation and rhythmic firing in rat subthalamic neurons. J. Neurosci..

[bb0035] Bevan M.D., Magill P.J., Terman D., Bolam J.P., Wilson C.J. (2002). Move to the rhythm: oscillations in the subthalamic nucleus-external globus pallidus network. Trends Neurosci..

[bb0040] Brazhnik E., McCoy A.J., Novikov N., Hatch C.E., Walters J.R. (2016). Ventral medial thalamic nucleus promotes synchronization of increased high Beta oscillatory activity in the basal ganglia-thalamocortical network of the hemiparkinsonian rat. J. Neurosci..

[bb0045] Brittain J.S., Brown P. (2014). Oscillations and the basal ganglia: motor control and beyond. Neuroimage.

[bb0050] Brittain J.S., Sharott A., Brown P. (2014). The highs and lows of beta activity in cortico-basal ganglia loops. Eur. J. Neurosci..

[bb0055] Buzsaki G., Anastassiou C.A., Koch C. (2012). The origin of extracellular fields and currents--EEG, ECoG, LFP and spikes. Nat. Rev. Neurosci..

[bb0060] Cagnan H., Mallet N., Moll C.K.E., Gulberti A., Holt A.B., Westphal M., Gerloff C., Engel A.K., Hamel W., Magill P.J., Brown P., Sharott A. (2019). Temporal evolution of beta bursts in the parkinsonian cortical and basal ganglia network. Proc. Natl. Acad. Sci. U. S. A..

[bb0065] Campbell P., Reep R.L., Stoll M.L., Ophir A.G., Phelps S.M. (2009). Conservation and diversity of Foxp2 expression in muroid rodents: functional implications. J. Comp. Neurol..

[bb0070] Cook R.D. (1977). Detection of influential observation in linear-regression. Technometrics.

[bb0075] Deffains M., Iskhakova L., Katabi S., Haber S.N., Israel Z., Bergman H. (2016). Subthalamic, not striatal, activity correlates with basal ganglia downstream activity in normal and parkinsonian monkeys. eLife.

[bb0080] Denker M., Timme M., Gruen S. (2008). Analyzing phase synchronization between neuronal spiking activity and local field potentials. Neurosci. Res..

[bb0085] Do M.T., Bean B.P. (2003). Subthreshold sodium currents and pacemaking of subthalamic neurons: modulation by slow inactivation. Neuron.

[bb0090] Engel A.K., Fries P. (2010). Beta-band oscillations--signalling the status quo?. Curr. Opin. Neurobiol..

[bb0095] Farries M.A., Kita H., Wilson C.J. (2010). Dynamic spike threshold and zero membrane slope conductance shape the response of subthalamic neurons to cortical input. J. Neurosci..

[bb0100] Feingold J., Gibson D.J., DePasquale B., Graybiel A.M. (2015). Bursts of beta oscillation differentiate postperformance activity in the striatum and motor cortex of monkeys performing movement tasks. Proc. Natl. Acad. Sci. U. S. A..

[bb0105] Fogelson N., Williams D., Tijssen M., van Bruggen G., Speelman H., Brown P. (2006). Different functional loops between cerebral cortex and the subthalmic area in Parkinson’s disease. Cerebral Cortex (New York, NY : 1991).

[bb0110] Hamel W., Fietzek U., Morsnowski A., Schrader B., Herzog J., Weinert D., Pfister G., Muller D., Volkmann J., Deuschl G., Mehdorn H.M. (2003). Deep brain stimulation of the subthalamic nucleus in Parkinson’s disease: evaluation of active electrode contacts. J. Neurol. Neurosurg. Psychiatry.

[bb0115] Holt A.B., Wilson D., Shinn M., Moehlis J., Netoff T.I. (2016). Phasic burst stimulation: a closed-loop approach to tuning deep brain stimulation parameters for Parkinson’s disease. PLoS Comput. Biol..

[bb0120] Holt A.B., Kormann E., Gulberti A., Potter-Nerger M., McNamara C.G., Cagnan H., Baaske M.K., Little S., Koppen J.A., Buhmann C., Westphal M., Gerloff C., Engel A.K., Brown P., Hamel W., Moll C.K.E., Sharott A. (2019). Phase-dependent suppression of Beta oscillations in Parkinson’s disease patients. J. Neurosci..

[bb0125] Hontanilla B., Parent A., Gimenez-Amaya J.M. (1997). Parvalbumin and calbindin D-28k in the entopeduncular nucleus, subthalamic nucleus, and substantia nigra of the rat as revealed by double-immunohistochemical methods. Synapse.

[bb0130] Janssen M.L.F., Temel Y., Delaville C., Zwartjes D.G.M., Heida T., De Deurwaerdere P., Visser-Vandewalle V., Benazzouz A. (2017). Cortico-subthalamic inputs from the motor, limbic, and associative areas in normal and dopamine-depleted rats are not fully segregated. Brain Struct. Funct..

[bb0135] Kempf F., Brucke C., Salih F., Trottenberg T., Kupsch A., Schneider G.H., Doyle Gaynor L.M., Hoffmann K.T., Vesper J., Wohrle J., Altenmuller D.M., Krauss J.K., Mazzone P., Di Lazzaro V., Yelnik J., Kuhn A.A., Brown P. (2009). Gamma activity and reactivity in human thalamic local field potentials. Eur. J. Neurosci..

[bb0140] Khanna P., Carmena J.M. (2015). Neural oscillations: beta band activity across motor networks. Curr. Opin. Neurobiol..

[bb0145] Khanna P., Carmena J.M. (2017). Beta band oscillations in motor cortex reflect neural population signals that delay movement onset. Elife.

[bb0150] Kita H., Kita T. (2011). Role of striatum in the pause and burst generation in the globus pallidus of 6-OHDA-treated rats. Front. Syst. Neurosci..

[bb0155] Kuhn A.A., Trottenberg T., Kivi A., Kupsch A., Schneider G.H., Brown P. (2005). The relationship between local field potential and neuronal discharge in the subthalamic nucleus of patients with Parkinson’s disease. Exp. Neurol..

[bb0160] Kuhn A.A., Kempf F., Brucke C., Gaynor Doyle L., Martinez-Torres I., Pogosyan A., Trottenberg T., Kupsch A., Schneider G.H., Hariz M.I., Vandenberghe W., Nuttin B., Brown P. (2008). High-frequency stimulation of the subthalamic nucleus suppresses oscillatory beta activity in patients with Parkinson’s disease in parallel with improvement in motor performance. J. Neurosci..

[bb0165] Kühn A.A., Tsui A., Aziz T., Ray N., Brucke C., Kupsch A., Schneider G.H., Brown P. (2009). Pathological synchronisation in the subthalamic nucleus of patients with Parkinson’s disease relates to both bradykinesia and rigidity. Exp. Neurol..

[bb0170] Lachaux J.P., Rodriguez E., Martinerie J., Varela F.J. (1999). Measuring phase synchrony in brain signals. Hum. Brain Mapp..

[bb0175] Lalo E., Thobois S., Sharott A., Polo G., Mertens P., Pogosyan A., Brown P. (2008). Patterns of bidirectional communication between cortex and basal ganglia during movement in patients with Parkinson disease. J. Neurosci..

[bb0180] Levy R., Ashby P., Hutchison W.D., Lang A.E., Lozano A.M., Dostrovsky J.O. (2002). Dependence of subthalamic nucleus oscillations on movement and dopamine in Parkinson’s disease. Brain J. Neurol..

[bb0185] Little S., Pogosyan A., Kuhn A.A., Brown P. (2012). Beta band stability over time correlates with parkinsonian rigidity and bradykinesia. Exp. Neurol..

[bb0190] Little S., Pogosyan A., Neal S., Zavala B., Zrinzo L., Hariz M., Foltynie T., Limousin P., Ashkan K., FitzGerald J., Green A.L., Aziz T.Z., Brown P. (2013). Adaptive deep brain stimulation in advanced Parkinson disease. Ann. Neurol..

[bb0195] Lofredi R., Neumann W.J., Bock A., Horn A., Huebl J., Siegert S., Schneider G.H., Krauss J.K., Kuhn A.A. (2018). Dopamine-dependent scaling of subthalamic gamma bursts with movement velocity in patients with Parkinson’s disease. Elife.

[bb0200] Magill P.J., Bolam J.P., Bevan M.D. (2000). Relationship of activity in the subthalamic nucleus-globus pallidus network to cortical electroencephalogram. J. Neurosci..

[bb0205] Magill P.J., Bolam J.P., Bevan M.D. (2001). Dopamine regulates the impact of the cerebral cortex on the subthalamic nucleus-globus pallidus network. Neuroscience.

[bb0210] Magill P.J., Sharott A., Bevan M.D., Brown P., Bolam J.P. (2004). Synchronous unit activity and local field potentials evoked in the subthalamic nucleus by cortical stimulation. J. Neurophysiol..

[bb0215] Mallet N., Ballion B., Le Moine C., Gonon F. (2006). Cortical inputs and GABA interneurons imbalance projection neurons in the striatum of parkinsonian rats. J. Neurosci..

[bb0220] Mallet N., Pogosyan A., Marton L.F., Bolam J.P., Brown P., Magill P.J. (2008). Parkinsonian beta oscillations in the external globus pallidus and their relationship with subthalamic nucleus activity. J. Neurosci..

[bb0225] Mallet N., Pogosyan A., Sharott A., Csicsvari J., Bolam J.P., Brown P., Magill P.J. (2008). Disrupted dopamine transmission and the emergence of exaggerated beta oscillations in subthalamic nucleus and cerebral cortex. J. Neurosci..

[bb0230] Mallet N., Micklem B.R., Henny P., Brown M.T., Williams C., Bolam J.P., Nakamura K.C., Magill P.J. (2012). Dichotomous organization of the external globus pallidus. Neuron.

[bb0235] Marsden J.F., Limousin-Dowsey P., Ashby P., Pollak P., Brown P. (2001). Subthalamic nucleus, sensorimotor cortex and muscle interrelationships in Parkinson’s disease. Brain.

[bb0240] McNamara C.G., Rothwell M., Sharott A. (2020). Phase-dependent closed-loop modulation of neural oscillations in vivo. bioRxiv.

[bb0245] Meidahl A.C., Tinkhauser G., Herz D.M., Cagnan H., Debarros J., Brown P. (2017). Adaptive deep brain stimulation for movement disorders: the long road to clinical therapy. Mov. Disord..

[bb0250] Moll C.K., Galindo-Leon E., Sharott A., Gulberti A., Buhmann C., Koeppen J.A., Biermann M., Baumer T., Zittel S., Westphal M., Gerloff C., Hamel W., Munchau A., Engel A.K. (2014). Asymmetric pallidal neuronal activity in patients with cervical dystonia. Front. Syst. Neurosci..

[bb0255] Moran A., Bergman H., Israel Z., Bar-Gad I. (2008). Subthalamic nucleus functional organization revealed by parkinsonian neuronal oscillations and synchrony. Brain J. Neurol..

[bb0260] Murthy V.N., Fetz E.E. (1992). Coherent 25- to 35-Hz oscillations in the sensorimotor cortex of awake behaving monkeys. Proc. Natl. Acad. Sci. U. S. A..

[bb0265] Murthy V.N., Fetz E.E. (1996). Oscillatory activity in sensorimotor cortex of awake monkeys: synchronization of local field potentials and relation to behavior. J. Neurophysiol..

[bb0270] Nakamura K.C., Sharott A., Magill P.J. (2014). Temporal coupling with cortex distinguishes spontaneous neuronal activities in identified basal ganglia-recipient and cerebellar-recipient zones of the motor thalamus. Cereb. Cortex.

[bb0275] Nakanishi H., Kita H., Kitai S.T. (1987). Electrical membrane properties of rat subthalamic neurons in an in vitro slice preparation. Brain Res..

[bb0280] Nambu A., Tokuno H., Hamada I., Kita H., Imanishi M., Akazawa T., Ikeuchi Y., Hasegawa N. (2000). Excitatory cortical inputs to pallidal neurons via the subthalamic nucleus in the monkey. J. Neurophysiol..

[bb0285] Nambu A., Tokuno H., Takada M. (2002). Functional significance of the cortico-subthalamo-pallidal ‘hyperdirect’ pathway. Neurosci. Res..

[bb0290] Neumann W.J., Degen K., Schneider G.H., Brucke C., Huebl J., Brown P., Kuhn A.A. (2016). Subthalamic synchronized oscillatory activity correlates with motor impairment in patients with Parkinson’s disease. Mov. disord..

[bb0295] Oswal A., Beudel M., Zrinzo L., Limousin P., Hariz M., Foltynie T., Litvak V., Brown P. (2016). Deep brain stimulation modulates synchrony within spatially and spectrally distinct resting state networks in Parkinson’s disease. Brain J. Neurol..

[bb0300] Pasquereau B., Turner R.S. (2011). Primary motor cortex of the parkinsonian monkey: differential effects on the spontaneous activity of pyramidal tract-type neurons. Cereb. Cortex.

[bb0305] Pinault D. (1996). A novel single-cell staining procedure performed in vivo under electrophysiological control: morpho-functional features of juxtacellularly labeled thalamic cells and other central neurons with biocytin or Neurobiotin. J. Neurosci. Methods.

[bb0310] Priori A., Foffani G., Pesenti A., Tamma F., Bianchi A.M., Pellegrini M., Locatelli M., Moxon K.A., Villani R.M. (2004). Rhythm-specific pharmacological modulation of subthalamic activity in Parkinson’s disease. Exp. Neurol..

[bb0315] Quiroga-Varela A., Walters J.R., Brazhnik E., Marin C., Obeso J.A. (2013). What basal ganglia changes underlie the parkinsonian state? The significance of neuronal oscillatory activity. Neurobiol. Dis..

[bb0320] Rivlin-Etzion M., Ritov Y., Heimer G., Bergman H., Bar-Gad I. (2006). Local shuffling of spike trains boosts the accuracy of spike train spectral analysis. J. Neurophysiol..

[bb0325] Rosin B., Slovik M., Mitelman R., Rivlin-Etzion M., Haber S.N., Israel Z., Vaadia E., Bergman H. (2011). Closed-loop deep brain stimulation is superior in ameliorating parkinsonism. Neuron.

[bb0452] Schindelin J, Arganda-Carreras I, Frise E, Kaynig V, Longair M, Pietzsch T, Preibisch S, Rueden C, Saalfeld S, Schmid B, Tinevez JY, White DJ, Hartenstein V, Eliceiri K, Tomancak P, Cardona A (2012) Fiji: an open-source platform for biological-image analysis. Nature methods 9:676-682. 10.1038/nmeth.2019 PMC3855844 22743772

[bb0330] Sharott A., Magill P.J., Harnack D., Kupsch A., Meissner W., Brown P. (2005). Dopamine depletion increases the power and coherence of beta-oscillations in the cerebral cortex and subthalamic nucleus of the awake rat. Eur. J. Neurosci..

[bb0335] Sharott A., Doig N.M., Mallet N., Magill P.J. (2012). Relationships between the firing of identified striatal interneurons and spontaneous and driven cortical activities in vivo. J. Neurosci..

[bb0340] Sharott A., Gulberti A., Zittel S., Tudor Jones A.A., Fickel U., Munchau A., Koppen J.A., Gerloff C., Westphal M., Buhmann C., Hamel W., Engel A.K., Moll C.K. (2014). Activity parameters of subthalamic nucleus neurons selectively predict motor symptom severity in Parkinson’s disease. J. Neurosci..

[bb0345] Sharott A., Vinciati F., Nakamura K.C., Magill P.J. (2017). A population of indirect pathway striatal projection neurons is selectively entrained to parkinsonian Beta oscillations. J. Neurosci..

[bb0350] Sharott A., Gulberti A., Hamel W., Koppen J.A., Munchau A., Buhmann C., Potter-Nerger M., Westphal M., Gerloff C., Moll C.K.E., Engel A.K. (2018). Spatio-temporal dynamics of cortical drive to human subthalamic nucleus neurons in Parkinson’s disease. Neurobiol. Dis..

[bb0355] Sherman M.A., Lee S., Law R., Haegens S., Thorn C.A., Hamalainen M.S., Moore C.I., Jones S.R. (2016). Neural mechanisms of transient neocortical beta rhythms: converging evidence from humans, computational modeling, monkeys, and mice. Proc. Natl. Acad. Sci. U. S. A..

[bb0360] Shimamoto S.A., Ryapolova-Webb E.S., Ostrem J.L., Galifianakis N.B., Miller K.J., Starr P.A. (2013). Subthalamic nucleus neurons are synchronized to primary motor cortex local field potentials in Parkinson’s disease. J. Neurosci..

[bb0365] Shin H., Law R., Tsutsui S., Moore C.I., Jones S.R. (2017). The rate of transient beta frequency events predicts behavior across tasks and species. Elife.

[bb0370] Smith Y., Bevan M.D., Shink E., Bolam J.P. (1998). Microcircuitry of the direct and indirect pathways of the basal ganglia. Neuroscience.

[bb0375] Stein E., Bar-Gad I. (2012). Beta oscillations in the cortico-basal ganglia loop during parkinsonism. Exp. Neurol..

[bb0380] Sterio D., Zonenshayn M., Mogilner A.Y., Rezai A.R., Kiprovski K., Kelly P.J., Beric A. (2002). Neurophysiological refinement of subthalamic nucleus targeting. Neurosurgery.

[bb0385] Tachibana Y., Iwamuro H., Kita H., Takada M., Nambu A. (2011). Subthalamo-pallidal interactions underlying parkinsonian neuronal oscillations in the primate basal ganglia. Eur. J. Neurosci..

[bb0390] Tinkhauser G., Pogosyan A., Tan H., Herz D.M., Kuhn A.A., Brown P. (2017). Beta burst dynamics in Parkinson’s disease OFF and ON dopaminergic medication. Brain J. Neurol..

[bb0395] Tinkhauser G., Pogosyan A., Little S., Beudel M., Herz D.M., Tan H., Brown P. (2017). The modulatory effect of adaptive deep brain stimulation on beta bursts in Parkinson’s disease. Brain J. Neurol..

[bb0400] Tinkhauser G., Torrecillos F., Duclos Y., Tan H., Pogosyan A., Fischer P., Carron R., Welter M.L., Karachi C., Vandenberghe W., Nuttin B., Witjas T., Regis J., Azulay J.P., Eusebio A., Brown P. (2018). Beta burst coupling across the motor circuit in Parkinson’s disease. Neurobiol. Dis..

[bb0405] Torrecillos F., Tinkhauser G., Fischer P., Green A.L., Aziz T.Z., Foltynie T., Limousin P., Zrinzo L., Ashkan K., Brown P., Tan H. (2018). Modulation of beta bursts in the subthalamic nucleus predicts motor performance. J. Neurosci..

[bb0410] Vinck M., van Wingerden M., Womelsdorf T., Fries P., Pennartz C.M. (2010). The pairwise phase consistency: a bias-free measure of rhythmic neuronal synchronization. NeuroImage.

[bb0415] Watson C., Paxinos G. (1986). The Rat Brain in Stereotaxic Coordinates.

[bb0420] West T., Farmer S., Berthouze L., Jha A., Beudel M., Foltynie T., Limousin P., Zrinzo L., Brown P., Litvak V. (2016). The parkinsonian subthalamic network: measures of power, linear, and non-linear synchronization and their relationship to L-DOPA treatment and OFF state motor severity. Front. Hum. Neurosci..

[bb0425] Williams D., Tijssen M., Van Bruggen G., Bosch A., Insola A., Di Lazzaro V., Mazzone P., Oliviero A., Quartarone A., Speelman H., Brown P. (2002). Dopamine-dependent changes in the functional connectivity between basal ganglia and cerebral cortex in humans. Brain J. Neurol..

[bb0430] Wilson C.J. (2013). Active decorrelation in the basal ganglia. Neuroscience.

[bb0435] Wilson C.J. (2015). Oscillators and oscillations in the basal ganglia. Neuroscientist.

[bb0440] Zaidel A., Arkadir D., Israel Z., Bergman H. (2009). Akineto-rigid vs. tremor syndromes in parkinsonism. Curr. Opin. Neurol..

[bb0445] Zaidel A., Spivak A., Grieb B., Bergman H., Israel Z. (2010). Subthalamic span of beta oscillations predicts deep brain stimulation efficacy for patients with Parkinson’s disease. Brain J. Neurol..

[bb0450] Zold C.L., Escande M.V., Pomata P.E., Riquelme L.A., Murer M.G. (2012). Striatal NMDA receptors gate cortico-pallidal synchronization in a rat model of Parkinson’s disease. Neurobiol. Dis..

