# Supplementary material for: Parkinson's disease uncovers an underlying sensitivity of subthalamic nucleus neurons to beta-frequency cortical input in vivo
Source: Neurobiol Dis. 2020 Dec;146:105119. doi: 10.1016/j.nbd.2020.105119 (PMC7710979; doi:10.1016/j.nbd.2020.105119)
Supplement: Supplementary file 1 — Supplementary material [file mmc1.pdf]

Supplementary Material

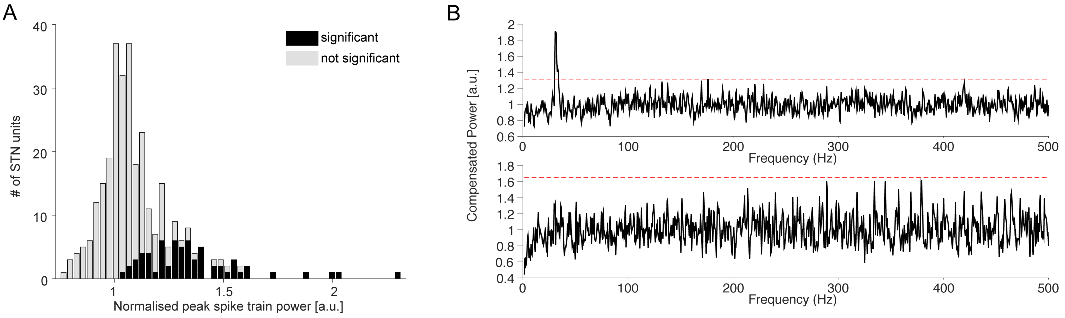

Supplementary Figure 1. Distribution of subthalamic oscillatory spike train power at beta peak frequency. A, Histogram showing the normalised compensated spike train power of the highest peak in the beta frequency range (12-40 Hz) normalised by the compensated power from 60-90 Hz (n=302 STN units (all available units regardless of phase-locking behaviour), STN units showing significant oscillations n=64, STN units without significant oscillations n=238). The compensated power is obtained by dividing the original power spectrum by the power spectrum of 100 shuffled spike trains. Significant oscillations are determined by a peak reaching power values above the 5-95% confidence interval of the power between 300-500 Hz. Grey bars show the beta power of spike trains without significant oscillations, while black bars show the beta power of spike trains with significant oscillations based on these criteria. **B**, Example compensated power spectra of an STN unit showing significant oscillatory activity around 30 Hz (top) and a STN unit showing no significant oscillatory activity (bottom).

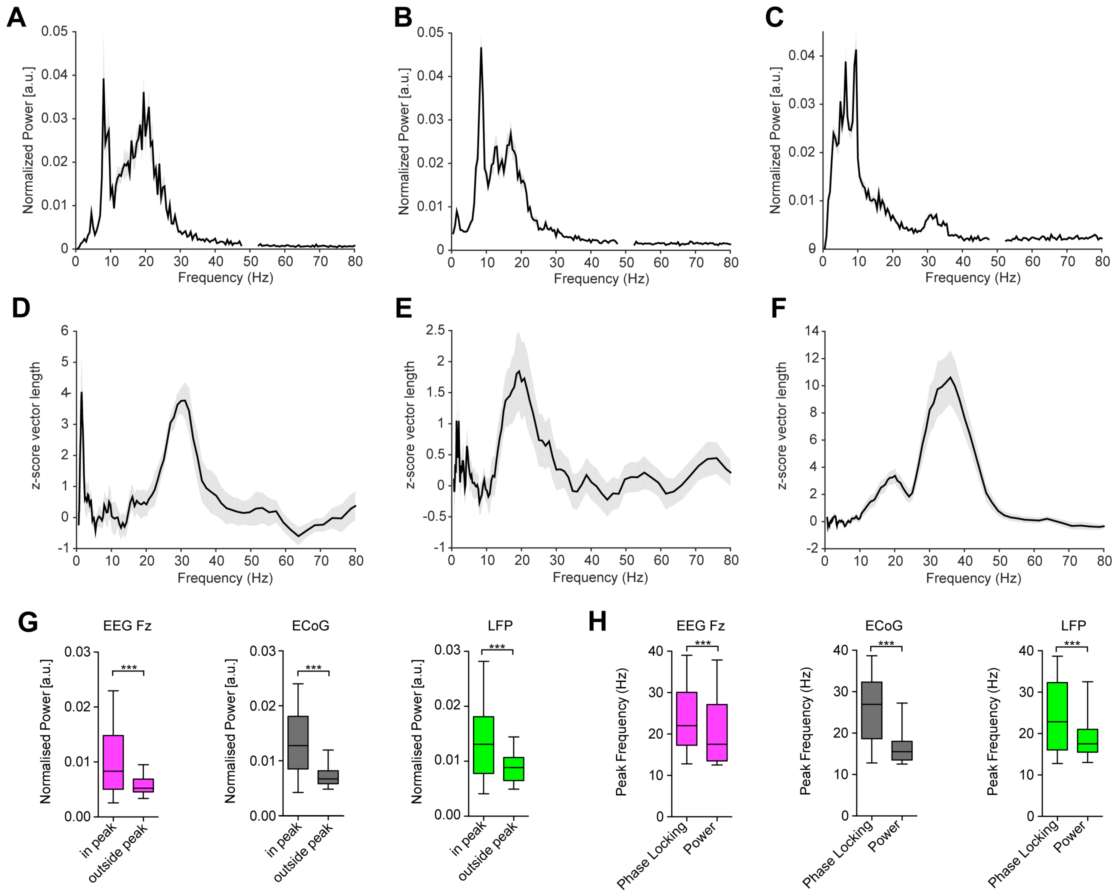

Supplementary Figure 2. Description of peak frequency of averaged ECoG/EEG/LFP power spectra and phase-locking. A-C, Averaged normalised power spectra of ECoG recordings from 3 example hemispheres from 3 different patients. Please note the different individual peak frequencies for each patient (A, n=9 unit-ECoG pairs; B, n=15 unit-ECoG pairs; C, n=22 unit-ECoG pairs). **D-F**, Z-score vector length across frequencies of the phase locking analysis of STN units to ECoG recordings from the same hemispheres shown in A-C. Please note that A and D, B and E, C and F are obtained from the same hemisphere/patient. The examples show that the peak frequency is different in individuals and moreover the peak frequency in the average power spectrum does not necessarily reflect the frequency of preferred locking. **G**, Statistical comparison between peak of z-score vector length (labelled Phase Locking) and power (normalised power [a.u.]) of the network population signal (Fz (pink, n=154), ECoG (grey, n=109) and LFP (green, n=172) in the frequency range between 12-40 Hz. Please note that the peak frequency of phase-locking is slightly higher for the phase-locking analysis in comparison to the peak frequency of the power spectra. **H**, Statistical comparison of power of the network population signal (Fz (pink), ECoG (grey) and LFP (green)) at the frequency of highest phaselocking (+/- 2 Hz) and around that frequency (bins between 12-40 Hz included with exception of the peak +/- 2 Hz). Please note that the power in the averaged power spectra is significantly higher at the frequency of preferred locking in comparison to outside that peak. For statistical comparison in G and H Wilcoxon rank test is used. Box plots show the quartile boundaries with whiskers showing the 5-95 percentiles, $p<0.001$ ***.

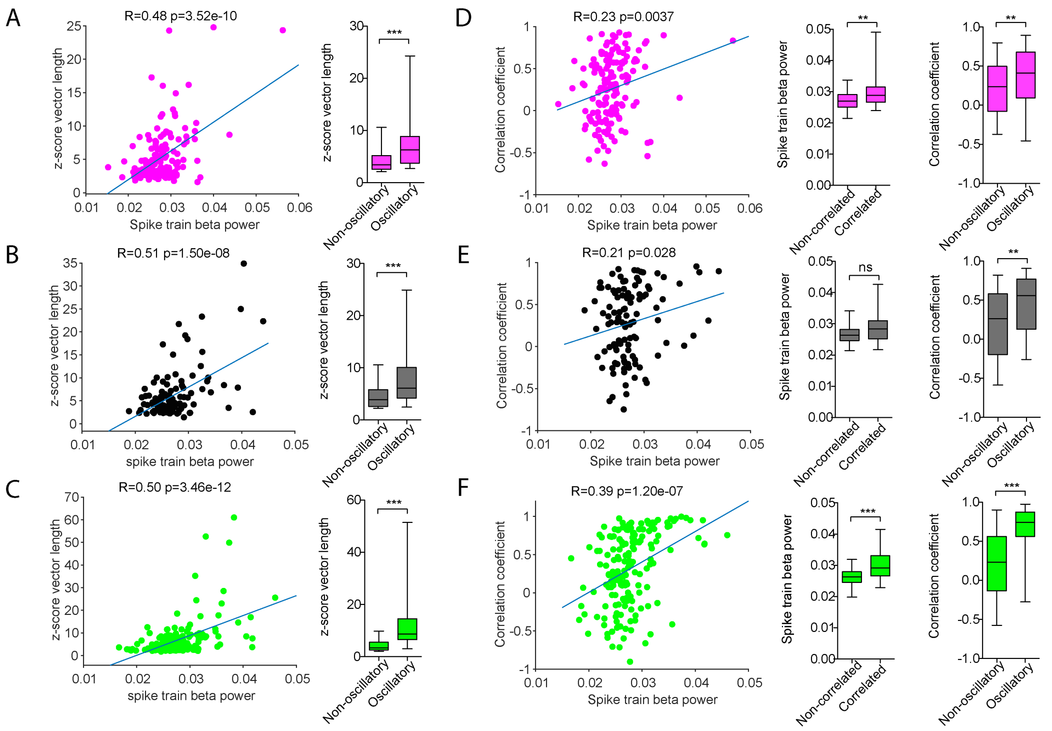

Supplementary Figure 3. Phase-locking and entrainment of STN units to beta oscillations is associated with a higher oscillation strength of STN units. Complementary analysis of the relationship between spike train oscillation power and metrics of phase-locking referring to Fig. 4 of the main manuscript. **A-C,** Dots in the scatter plot show the mean z-score vector length (phase-locking strength) at the beta frequency of preferred phase-locking (12-40 Hz) and spike train beta power at the same frequency +/- 5 Hz normalised by the spike train power in the range 300-500 Hz for each recorded pair. The phase-locking strength is significantly positively correlated with the normalized spike train power for all investigated signals (Fz Pearson's R=0.48, *p*=3.52e-10; ECoG Pearson's R=0.51, *p*=1.50e-08; LFP Pearson's R=0.50, *p*=3.46e-12). The line indicates the linear fit of the correlation. The boxplots on the right hand side show the z-score vector length for non-oscillatory and oscillatory units with a significant higher phase-locking strength for oscillatory units for all investigated population oscillation signals (Fz (A), ECoG (B) and LFP (C). (Fz: non-oscillatory units (n=106/154), oscillatory units (n=48/154), *p*=1.82e-06; ECoG: non-oscillatory units (n=70/109), oscillatory units (n=39/109), *p=8*.32e-05; LFP: non-oscillatory units (n=125/172), oscillatory units (n=47/172), *p=1.39*e-12)). **D-F,** Show the relationship between entrainment of STN unit to beta oscillations (measured by the Pearson's correlation coefficient between the phase-locking strength and the mean magnitude at each percentile) and oscillatory properties of the STN units for all population oscillation signals EEG Fz (D), ECoG (E) and LFP (F). Left, Scatterplots show the Pearson's correlation between the Pearson's correlation coefficient (z-score vector length x mean normalised magnitude at each percentile) and the spike train power in the preferred frequency of phase-locking +/- 5 Hz analogue to A-C. Note the significant positive correlation between magnitude-dependent phase-locking and oscillation strength for pairs with all population oscillation signals (Fz Pearson's R=0.23,

p=0.004; ECoG Pearson's R=0.21, *p*=0.028; LFP Pearson's R=0.39, *p*=1.20e-07). Middle, Boxplots show that the normalized spike train beta power (as described above) is higher for significantly positive correlated unit-field pairs (Fz: non-correlated units (n=126/154), magnitude-correlated units (n=28/154), *p*=0.007; ECoG: non-correlated units (n=82/109), magnitude-correlated units (n=27/109), *p=0.11*; LFP: non-correlated units (n=116/172), magnitude-correlated units (n=56/172), *p=2.67*e-07). Right, Boxplots show the Pearson's correlation coefficient (z-score vector length x mean normalised magnitude at percentile) for non-oscillatory and oscillatory units (Fz: non-oscillatory units (n=106/154), oscillatory units (n=48/154), *p*=0.007; ECoG: non-oscillatory units (n=70/109), oscillatory units (n=39/109), *p=0.002*; LFP: non-oscillatory units (n=125/172), oscillatory units (n=47/172), *p=1.10*e-07). This indicates, that oscillatory neurons follow more strongly and likely the magnitude of beta oscillations. Pairwise comparisons were performed using MWUT. *** p<0.001, **p<0.01, *p<0.05, whiskers of boxplots show the 5-95th percentile.

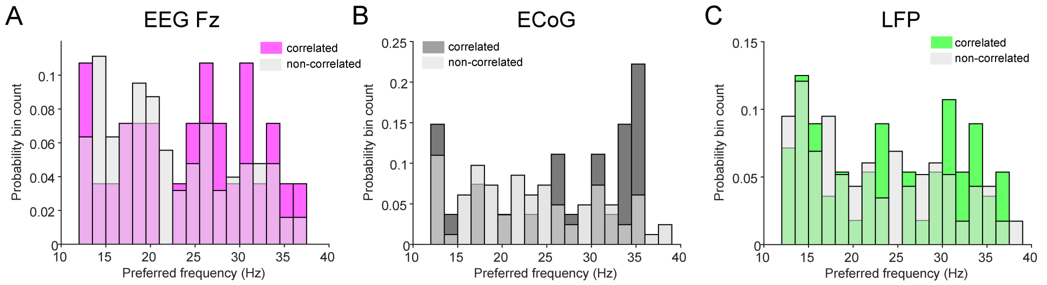

Supplementary Figure 4. Preferred frequencies of phase-locking. Fig. 4 of the main manuscript shows metrics describing the relationship between magnitude and phase-locking only in the beta frequency range. This figure shows the frequency of preferred locking in the beta-frequency range chosen for further analyses shown in Fig. 4 of the main manuscript. Please note that frequencies of preferred phase-locking were considered in the calculation between the frequencies of 12 and 40 Hz, but in fact neurons tended to prefer significant phase-locking in the frequencies 12 to 35 Hz. Intermediate colours indicate the overlap in preferred frequencies of locking for correlated and non-correlated neurons. **A-C,** Comparison of preferred frequencies of phase-locking between magnitude-correlated and non-correlated (correlation between z-score vector length (phase-locking strength) and mean normalised magnitude in each percentile) phase-locking pairs for Fz (A), ECoG (B) and LFP (C). Note that only the frequency range from 12-40 Hz was considered and the preferred frequency was defined as the frequency with the lowest p-value of the Raileigh-test. Data show that the preferred frequency of phase-locking to beta oscillations is widely distributed and is not different between units that get entrained by the magnitude of population oscillations and those which do not (Fz: non-correlated units (n=126): 23.26 +/- 7.79 Hz, magnitude-correlated (n=28): 24.31 +/- 7.43 Hz, MWUT p=0.43; ECoG: non-correlated units (n=82): 24.03 +/- 7.76 Hz, magnitude-correlated (n=27): 26.69 +/- 8.53 Hz, MWUT p=0.11; LFP: non-correlated units (n=56): 22.76 +/- 7.8 Hz, magnitude-correlated (n=53): 24.10 +/- 8.04 Hz, MWUT p=0.27).

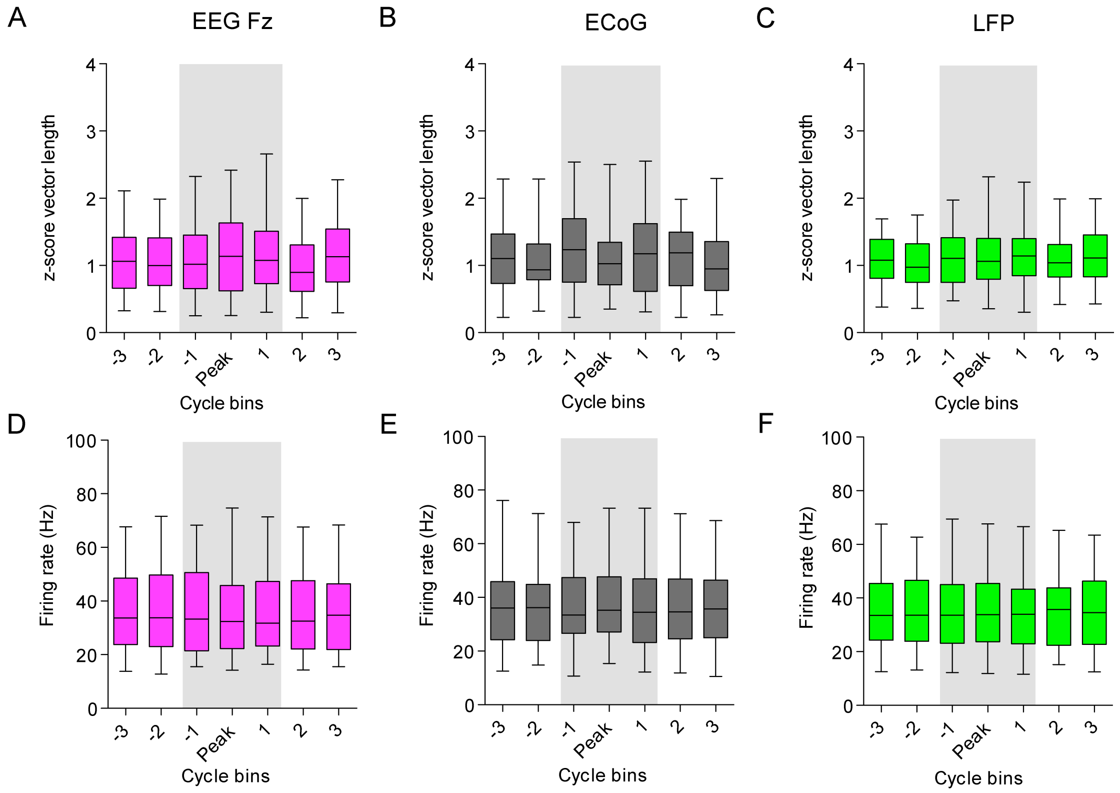

Supplementary Figure 5. A subset of STN-neurons is not entrained during epochs elevated beta power (beta bursts). A-C, Beta burst analysis of STN-units, whose phase-locking strength is not significantly correlated with the magnitude of the ongoing population oscillation signal. Phase-locking analysis during episodes of elevated beta power detected with a threshold at the 75th percentile of the magnitude for EEG Fz (A, pink, n=99), ECoG (B, grey, n=69) and LFP (C, green, n=94). Note that only STN unit-EEG/LFP pairs are shown which showed a negative or no correlation of phase-locking strength with the magnitude of the oscillation. STN unit-EEG/LFP pairs showing a positive significant correlation between phase-locking strength and magnitude of the oscillation are shown in Fig. 6 of the main manuscript. X-axis showing the averaged phase-locking strength of spikes in each cycle bin. The grey shaded area shows the phase-locking during a beta burst aligned to the peak of the beta burst. Only beta bursts with a minimum duration of 3 cycles of the preferred beta frequency were included. In case of a longer burst duration, those cycle bins are not shown in the figure. The bins -2 and -3 show the phase-locking outside a beta burst with a distance of one cycle to the start of the beta burst, so that -2 is the second cycle bin to the edge of the burst and -3 the 3rd cycle bin before the start of the beta burst. Analogue the bins 2 and 3 show the 2nd and 3rd cycle bin after the end of the beta burst. **D-E**, Mean firing rate in corresponding cycle bins showing in A-C for STN units during episodes of elevated beta power in EEG Fz (D), ECoG (E) and LFP (F). A Kruskal-Wallis Anova reveals no significant difference for phase-locking strength and firing rate within and outside beta bursts. Therefor neither the phase-locking strength nor the firing rate for this subset of STN neurons was modulated during beta bursts.

Supplementary Table 1. Patient details.

Case	Age (Yrs) and Sex	Disease Duration (Yrs)	Motor UPDRS OFF	Motor UPDRS ON	Medication pre-operation	Hoehn/ Yahr Score	Dominant side	Major Symptoms	Hemispheres analysed	ECoG
1	61F	25	50	30	Levodopa 700 mg<br>Carbidopa 150 mg<br>Entacapone 1000 mg<br>Benserazide 25 mg<br>Pramipexol 0.26 mg	5	Left	Tremor,<br>Akinesia/rigidity<br>Fluctuations	Left	Yes
2	70F	8	38	21	Ropinirole 8mg<br>Alpha-dihydroergocryptine 40 mg<br>Amantadine 600 mg<br>Levodopa 250mg<br>Carbidopa 150mg	3	Left	Bradykinesia<br>Fluctuations<br>Dyskinesia	Both	No
3	67M	25	55	19	Levodopa 1250 mg<br>Entacapone 1400 mg<br>Carbidopa 312.5mg<br>Rotigotine 6 mg<br>Amatadine 150mg	3	Right	Equivalence<br>Fluctuations<br>Dyskinesia	Both	Yes
4	64M	15	53	39	Amantadine 300 mg<br>Levodopa 550 mg<br>Ropinirole 20 mg<br>Entacapone 900 mg<br>Carbidopa 112.5 mg<br>Benserazide 25 mg	4	Left	Akinesia/rigidity<br>Camptocormia	Left	Yes
5	69F	9	21.5	7	Levodopa 450 mg<br>Lisuride 0.9 mg<br>Rotigotine 4 mg<br>Amantadine 300 mg	3	Right	Equivalence type<br>Fluctuations<br>Dyskinesia	Both	Yes

6	66M	11	28	14	Amantadine 150 mg<br>Levodopa 1450 mg<br>Tolcapone 300mg<br>Carbidopa 312.5<br>Benserazide 50mg	4	Right	Akinesia/rigidity<br>Fluctuations	Both	No
7	72F	18	41	19	Levodopa 700mg<br>Carbidopa 175mg	4	Left	Equivalence type<br>Fluctuations<br>Dyskinesia	Left	Yes
8	63F	17	35	16	Rotigotine 4mg<br>Ropinirole 25mg<br>Levodopa 350mg	3	Left	Akinesia/rigidity<br>Bradykinesia<br>Fluctuations<br>Dyskinesia	Both	Yes
9	56M	10	18	6	Amantadine 300 mg<br>Safinamide 100 mg<br>Levodopa 450 mg<br>Benserazide 112.5<br>Opicapone 50 mg<br>Pramipexole 2.1 mg	2	Left	Equivalence type<br>Fluctuations	Both	No
10	49M	10	21	8	Levodopa 725 mg<br>Entacapone 725 mg<br>Carbidopa 162.5 mg<br>Ropinirole 32 mg	2	Right	Bradykinesia<br>Akinesia/rigidity<br>Fluctuations	Both	No
11	66M	8	26	15	Levodopa 300 mg<br>Benserazide 75 mg<br>Rasagiline 1 mg	3	Right	Bradykinesia<br>Akinesia/rigidity<br>Fluctuations<br>Camptocormia	Both	No

12	74F	13	32	22	Amantadine 300 mg<br>Carbidopa 118.75 mg<br>Levodopa 575 mg<br>Rotigotine 14 mg	2-3	Left	Bradykinesia<br>Dyskinesia<br>Camptocormia	Both	Yes
13	58M	7	38	25	Amantadine 200 mg<br>Benserazide 30.5 mg<br>Levodopa 550 mg<br>Pramipexole 3.15 mg	2	Right	Equivalence type<br>Fluctuations	Both	No
14	53M	10	46	11	Benserazide 50 mg<br>Carbidopa 156.25 mg<br>Entacaopine 725 mg<br>Levodopa 825 mg<br>Ropinirole 16 mg	2-3	RIght	Equivalence type<br>Fluctuations	Both	Yes
15	57M	15	25	17	Amantadine 300 mg<br>Benserazide 125 mg<br>Levodopa 650 mg<br>Pramipexole 2.1 mg<br>Tolcapone 300 mg	2	Left	Bradykinesia<br>Akinesia/rigidity<br>Fluctuations<br>Dysarthrophonia	Right	Yes
16	57F	13	36	15	Amantadine 150 mg<br>Carbidopa 50 mg<br>Entacapone 200 mg<br>Levodopa 200 mg<br>Pramipexole 3.62 mg	2	Left	Bradykinesia<br>Akinesia/rigidity	Both	Yes
